# Natural based piperine derivatives as potent monoamine oxidase inhibitors: an in silico ADMET analysis and molecular docking studies

**DOI:** 10.1186/s13065-020-0661-0

**Published:** 2020-02-17

**Authors:** Priyanka Dhiman, Neelam Malik, Anurag Khatkar

**Affiliations:** 1 Department of Pharmaceutical Chemistry, Maharishi Markandeshwar College of Pharmacy, Maharishi Markandeshwar University, Mullana, Haryana 133203 India; 2 Laboratory for Preservation Technology and Enzyme Inhibition Studies, Faculty of Pharmaceutical Sciences, M. D. University, Rohtak, 124001 India

**Keywords:** Monoamine oxidase, In silico design, Piperine derivatives, DPPH, H_2_O_2_ activity

## Abstract

Neurodegenerative disorders follow numerous pathological ways concerning overexpression of monoamine oxidase and formation of reactive oxygen species. The computational design of the piperine derivatives has given the significant MAO inhibitors with considerable antioxidant potential. Molecular docking provided the mechanistic insight of the compounds within the hMAO active site. In the current study we have prepared a series of compounds related to piperine and investigated them through monoamine oxidase A and B assay and evaluated the free radical scavenging activity. The synthesized compounds were analyzed by using in silico techniques within the active site of MAO and the ADMET properties were also calculated. The results obtained in this study indicated the interesting therapeutic potential of some compounds such as **7**and **17c** as most promising hMAO-A inhibitors whereas compounds **15**, **5** and **17b** were found as hMAO-B inhibitors. Moreover, we assessed the antioxidant potential of the piperine analogues and compounds **5**, **17b**, and **7** showed very modest antioxidant activity against DPPH and H_2_O_2_ radicals. The outcome of the study indicating that the piperine related derivatives are found as considerable MAO inhibitors and antioxidants. Moreover, the SAR structure activity relationships are depicting the structural features required for the MAO inhibition. In case of MAO activity, good correlations were found among the calculated and experimental results.

## Background

**Fig. 1:**
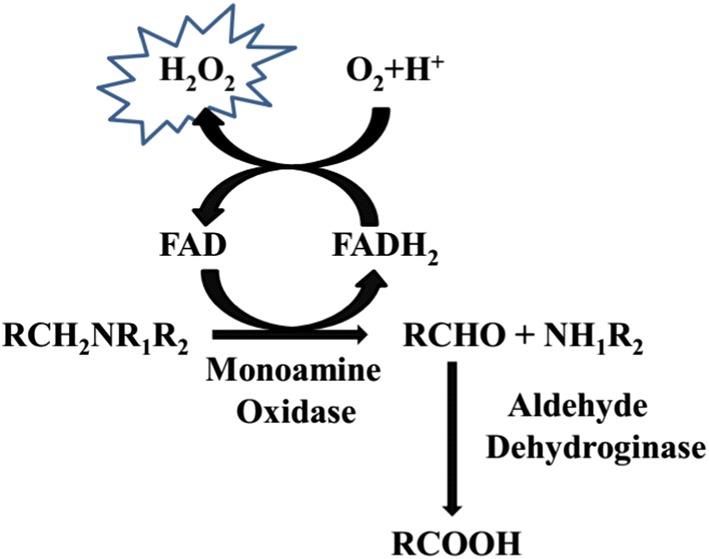
Graphic depiction of the oxidative deamination of monoaminergic neurotransmitter to corresponding aldehyde and hydrogen peroxide

The elementary nature of both monoamine oxidases (MAO, EC 1.4.3.4) isoforms (MAO-A and MAO-B) in the catabolism of monoaminergic neurotransmitters has been widely studied [1]. The two diverse isoforms, MAO-A and MAO-B, are categorized from their selectivity for the substrate and their specificity for the inhibitors. Flavin adenine dinucleotide (FAD) containing MAO found as placed on the mitochondrial outer membranes, and catalyzes the alpha-carbon oxidation of major monoamine neurotransmitter and modulating their levels in the peripheral tissues and brain [2]. MAO metabolizes the neurotransmitters and results in the production of reactive oxygen species like hydrogen peroxide and ammonia with corresponding aldehydes as neurotoxins (Fig. 1) [3]. This property of their modulation renders the brain signaling but overexpression of MAO leads to neurological imbalance, thus designing of the MAO inhibitors for the treatment of neuropsychiatric and neurodegenerative disorders, for instance, depression and Parkinson’s and Alzheimer’s diseases could be invaluable proposal [4].

Moreover, the disclosure of their 3D crystal structures based on their amino acid sequence and inhibitor sensitivity Binda et al. reported the crystal structures of hMAO-A and hMAO-B isoforms and highlighted their selective interaction with inhibitory ligands at the molecular level.

The crystallographic MAO structure comprised of three functional domains in the active core, called the entrance cavity, substrate cavity, and the “aromatic cage” [5]. This “aromatic cage” is created by Tyr435 and Tyr398 for MAO-A) whereas Tyr407 and Tyr444 for MAO-B with FAD (redox cofactor), and with some heterocyclic or aromatic compounds co-crystallized within an active site [6]. This information enthusiastically revived the interest of medicinal chemists to explore the rational design of selective and effective MAO inhibitors without undesirable side effects.

The appliance of molecular docking methods has enhanced the availability and identification of natural lead compounds [7]. Databases of natural products are widely mined for development of their target-based drug design by various screening approaches. Among all explored natural products piperine has been extensively studied for the MAO inhibition and has shown the significant potential to be an applicable candidate for neurological disorders [8].

Piperine (trans isomer of 1-piperolypiperidine) is an alkaloid found in the herb Black pepper (*Piper nigrum*) [9]. Recently, it was observed that phytochemicals extracted from the black pepper plant *Piper nigrum* were proficient to inhibit MAO-A and B [10]. Moreover, the docking calculations of the piperine inside the MAO active site reveals that the piperine establishes water-bridge formation with Cys172 and Tyr188, while an aromatic ring-hydrogen bond interaction was observed with Tyr398.

**Fig. 2:**
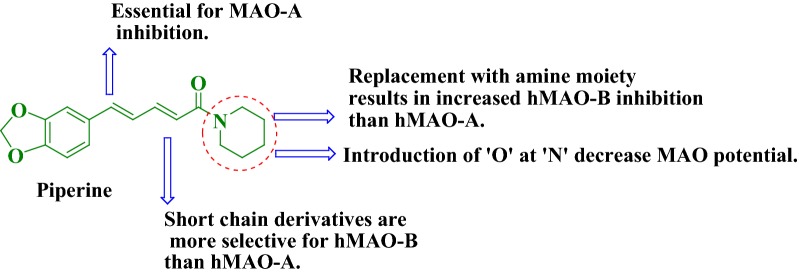
Reported pharmacophoric requirements on MAO activity of piperine

Another well-documented report also revealed that the structural water molecules of MAO-B active site interacted via hydrogen bonding with Cys 172 and Tyr 188 with the piperine [11]. In the case of MAO-A, the methylenedioxyphenyl ring established three hydrogen bonding interactions with water molecules of the hMAO-A active site. The piperine itself was surrounded by residues, for instance, Ile 180, Tyr 69, Ile 207, Gln 215, Asn 181, Ile 335, Tyr 407, Leu 337, Cys 323 along with FAD isoalloxazine moiety. Several reports have pointed out the essential structural features of piperine to be potent MAO inhibitor [12]. These features are summaries as followed (Fig. 2).

**Fig. 3:**
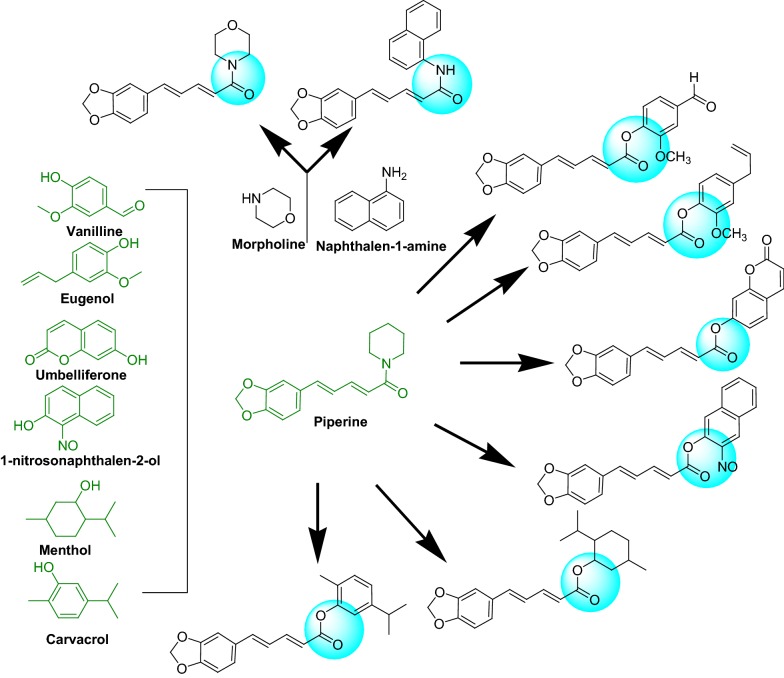
The design strategy for piperine based combinations

Encouraged by the aforementioned prerequisites, we synthesized and evaluated a series of piperine based derivative as hMAO inhibitors (Fig. 3). Moreover, the establishment of X-ray crystallographic structure information on MAO by Binda et al. prompted the medicinal chemists to computationally design the specific and effective MAO inhibitors using the pharmacophoric modifications and molecular docking [5]. The current study, based on the comparison of the dry lab and wet lab results of in silico designed and synthesized piperine derivatives and development a rational link for the selectivity of derivatives towards hMAO-A and hMAO-B isoforms. Additionally, the free radical scavenging activity was also investigated for antioxidant potential of titled compounds.

## Results

### Chemistry

**Scheme 1:**
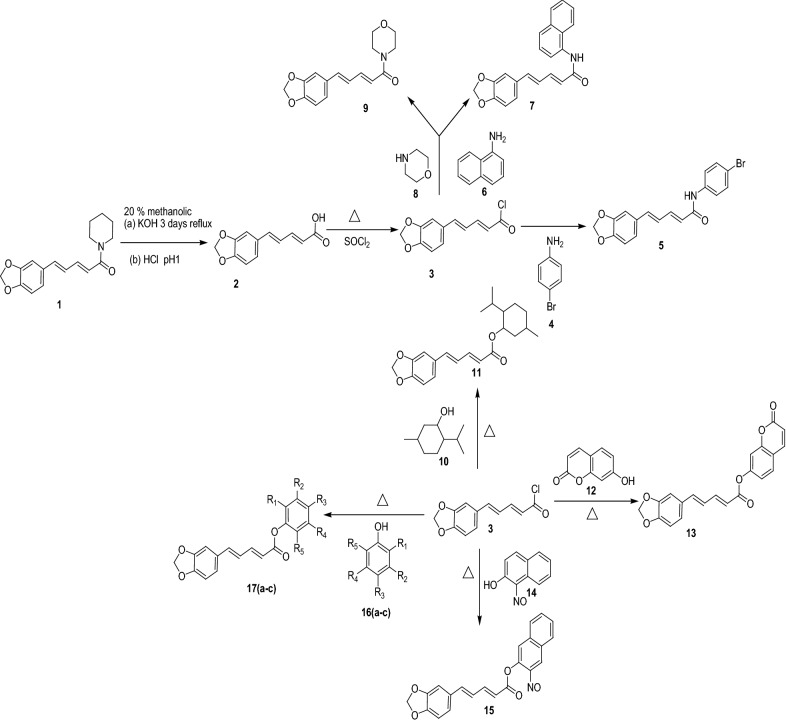
General schematic rout for the synthesis of novel piperine based derivatives

The procedures for the preparation of the targeted compounds **(5–17c)** are outlined in Scheme 1. Piperine **(1)**, commercially available vendor Hi-media and was converted into the acid **(2)** with 85% yield by the hydrolysis using KOH/EtOH for continuous reflux. An initial attempt to convert the acid **(2)** into the acid chloride **(3)** was carried out using thionyl chloride and followed by the extraction with dichloromethane and acetone/before the yields of the products were very low, and partial decomposition of the starting material was observed. So the addition of a few drops of pyridine during the above step yield better product without any decomposition. This tactic involving the use of pyridine was effectively useful for the synthesis of the acid chloride. Moreover, in the TLC, a single spot through R_f_ = 0.74 observed by a triple solvent system of ethyl hexane:toluene: ethyl acetate (1:1:1) for piperic acid chloride. The reaction progress was supervised through by IR spectra. Synthesis of the acyl chloride was definite subsequent wave number point in IR spectra peaks: carbonyl group confirm up approximately: 1684 cm^−1^ with the plain bond of OH group was noticed about 3448 cm^−1^ in the preparatory acid while the carbonyl of the acyl chloride shifted the peak around 1749 cm^−1^. Moreover, the disappearance of HNMR peak of piperidin-1-yl peak at 3.34 (singlet) and 1.50 (multiplet) while appearance of 11.0 (singlet) indicated the formation of piperic acid. Further in case of piperic acid chloride the 11.0 (singlet) was disappeared. The formation of multiplet at 7.61 indicated the formation of N-(4-bromophenyl) penta-2,4-dienamide bond of compound **5**. ^13^CNMR peaks at 123.17, 124.79, 131.44 indicated the N-(4-bromophenyl) penta-2,4-dienamide group formation of compound **5**. IR spectral peaks at 1648 cm^−1^ indicated the presence of 2^0^ amide and at 3009 cm^−1^ indicates aromatic stretch in compound **5**.

The piperic acid chloride was then converted into amides **(4**, **6**, **8)** as shown in Scheme 1 in the at 0–10 °C temperature. To prepare esters from acid chloride esterification of piperic acid chloride using natural phenols such as vanillin, eugenol, menthol, thymol, carvacrol and umbelliferone in acetone and dichloromethane as the solvent with a catalytic amount of H_2_SO_4_ under reflux afforded the natural-based hybrid esters **11**, **13**, **17a**–**17c** except **15**. IR spectra peak of esters derivatives was found to be more than 1857 cm^−1^ indicated the presence of ester group in **11**, **13**, **15**, **17a**–**17c** derivatives. Moreover, the doublet around 7.25 δ indicated the formation of ester linkage in compound **11**. ^13^CNMR peaks at 169.63 and 149.51 indicated the 5-(benzo[d] [1, 3] dioxol-5-yl)penta-2,4-dienoate linkage in compound **11**. In case of compound **15** IR spectra peak at 1860 cm^−1^ indicated the presence of ester group and ^1^HNMR (multiplet) peak at 7.63–7.53 indicated the incorporation of 3-nitrosonaphthalen-2-yl group at piperine. Moreover, the presence of ^13^CNMR peaks at 143.71 and 165.58 indicated the esteric linkage of 3-nitrosonaphthalen-2-yl group at piperine in compound **15**. IR spectra of **17a** was found as 3468 cm^−1^ for the phenol, 3039 aromatic stretch, 1710 for the ester, 1639 stretch for the alkene and 1218 for the C–O–C asymmetric stretch. Additionally, the elemental analysis and mass spectrometric data were also found in conformity with compounds characterization.

### MAO-A and MAO-B studies

The synthesized derivatives were screened for the hMAO-A and hMAO-B inhibitory activities by using an extremely sensitive continuous fluorometric assay containing Amplex Red (10-acetyl-3, 7-dihydroxyphenoxazine) reagent. The enzymatic H_2_O_2_ was measured with and without extracts or standards. The current study reveals that the two compounds **7** and **17c** were emerged as very modest hMAO-A inhibitors with IC_50_ values 15.38 ± 0.071 µM and 16.11 ± 0.091 µM, respectively. While the reference compounds clorgyline and piperine exhibited hMAO-A inhibitory potential by IC_50_ values of 18.74 ± 0.096 µM and 19.01 ± 0.031 µM, respectively.

In the case of hMAO-B compounds, **15** and **5** surprisingly shown the very modest hMAO-B inhibitory potential with IC_50_ values 12.15 ± 0.003 µM and 14.19 ± 0.007 µM, respectively plus good selectivity comparing with piperine. Although the reference compound pargyline and piperine revealed IC_50_ values for hMAO-B as 20.04 ± 0.095 µM and 17.57 ± 0.037 µM, respectively. The statistical significance: p < 0.05 against the equivalent IC_50_ values were achieved against MAO-A and MAO-B.

### Kinetic study of MAO inhibition

**Fig. 4:**
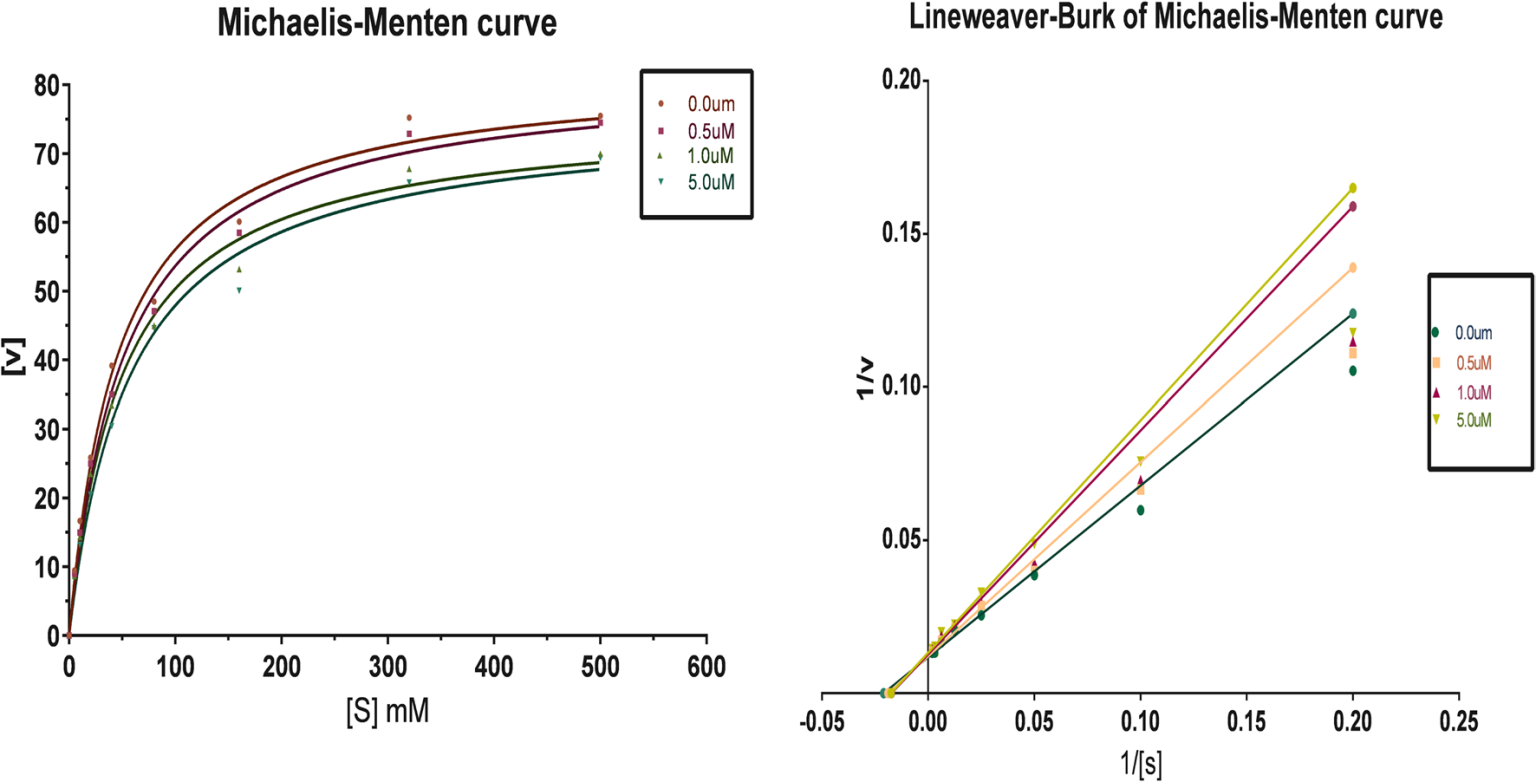
Kinetic study on the type of hMAO-A inhibition by compound **7**

**Fig. 5:**
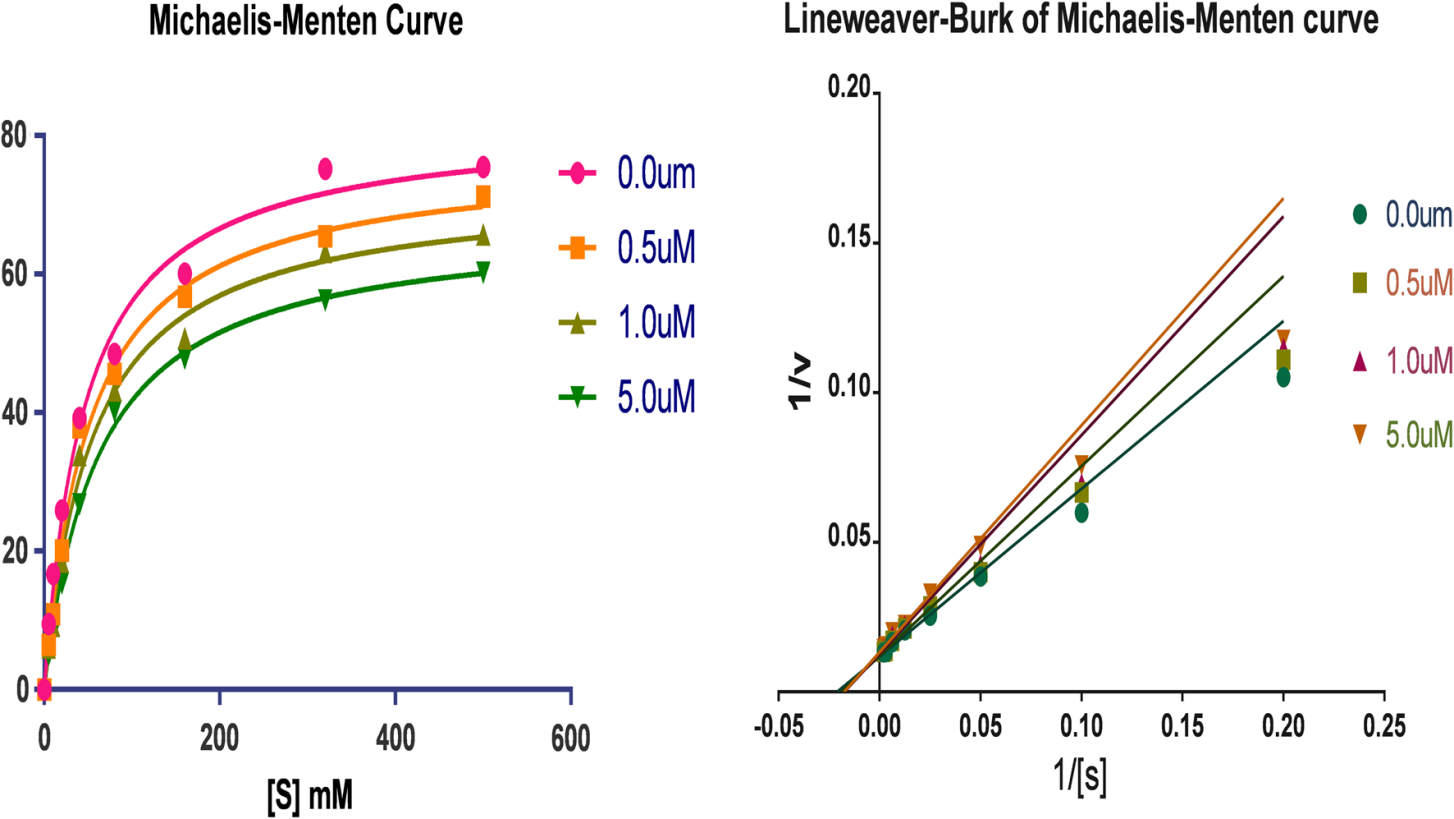
Kinetic study on the type of hMAO-B inhibition by compound **15**

The kinetic study by the Lineweaver–Burk plots, carried out to explicates the type of inhibition by compounds **7**, **15** shown their significant inhibitory potential towards hMAO-A and hMAO-B, respectively. Reaction mixtures composing of five diverse concentrations of p-tyramine was used as a common substrate in the presence or absence of compounds. The reciprocal Lineweaver–Burk plot attained reveals that both compounds **7**, **15** exhibited the same *V*_max_ rate at different concentrations; however, the value of *K*_m_ decreased with increasing concentration. Consequently, the inhibition of compounds **7**, **15** against hMAO-A and hMAO-B was specified to be competitive, as shown in Figs. 4, 5.

## Molecular modeling

**Fig. 6:**
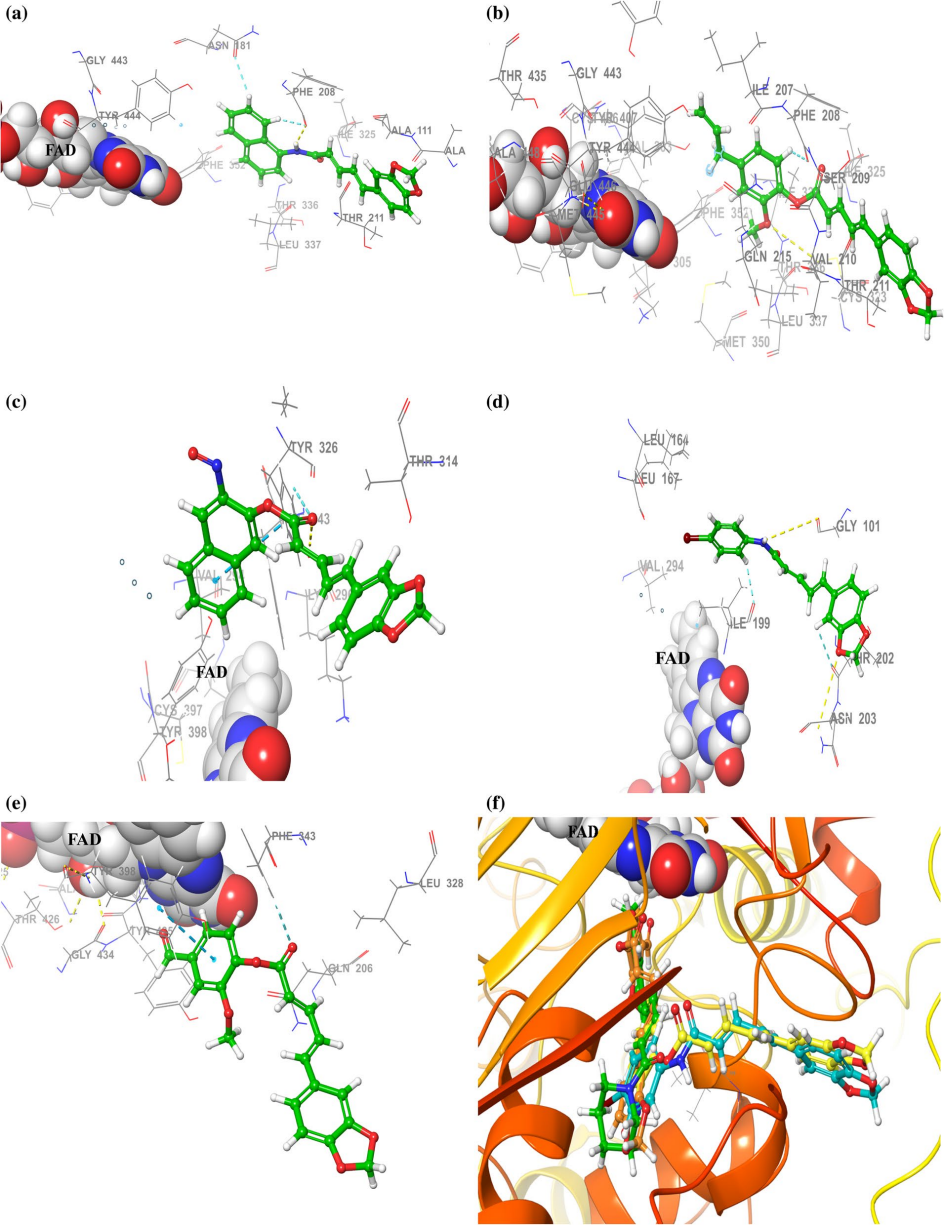
The binding modes of umbelliferone derivatives, **7** and **17c** in the active site of MAO-A is displayed as **a** and **b** respectively. Mode of the binding interaction of compounds **15**, **5**, **17b** within the MAO-B active site displayed as **c**–**e** respectively. Superimposed ligand structures of most active MAO-B inhibitory compounds **15**, **5**, **17b** within MAO-B cavity displayed in **f**)

To understand the structural basis for the mechanism of action of the piperine based compounds, molecular docking experiments were executed to calculate the binding modes of two most active compounds **7** and compound **17c** inside the MAO-A active site as shown in Fig. 6. The visual inspection regarding the putative orientation of the MAO-A inhibitor **7** showed the hydrogen bond is the most important residue Phe 208 through carbonyl group and NH group of dienamide linkage to the side chain. Close examination of the docking pose of compound **17c** into the active site of MAO-A depicted that the methoxyphenyl unit of the **17c** is located in the ‘aromatic cage’ outlined by Tyr407, Tyr197, and Tyr444, as shown in Fig. 6.

Conversely, in the case of hMAO-B compound **15** showed interesting binding orientations with the active site residues along with docking score of − 11.76. The nitrosonaphthalen-2-yl ring formed the π–π stacking bonds with Tyr326. Whereas the esteric carbonyl group established one hydrogen bond with the same residue Tyr 326, this simultaneous bridge formation may be responsible for the better binding affinity of compound **15** within the substrate domain. The graphical inspection afforded better details of the compound **5** which established two hydrogen bond interactions through the 2,4-dienamide group of **5**, one hydrogen bond between NH of 5 with Pro102 (OH) and another hydrogen bond with carbonyl oxygen and Tyr326 (C=O)of hMAO-B active site residues, as shown in Fig. 6.

Another notable hMAO-B inhibitor compound **17b** established π–π stacking interactions with Tyr398. The 2-methoxyphenyl unit of **17b** was emerged towards the FAD domain and was introduced into the ‘aromatic cage’ formulated by Phe168, Trp119, Phe103, and the aromatic ring. The side chain comprising conjugated double bonds was supported by ‘gatekeeper’ residue Ile199 and other hydrophobic residues Cys172 and Ile198. Methylenedioxyphenyl unit appeared as embedded in a large hydrophobic pocket framed by Pro102, Ile316, Phe103, Pro104, Trp119, Leu164, and Phe168. The 4-formyl-2-methoxyphenyl part of **17b** seemed as sandwiched between Phe343and Tyr188 on peripherals tough the ester linkage.

### In silico ADMET properties

In silico descriptors for the assessment of the pharmaceutically relevant properties, were calculated by QikProp interface and various parameters such as Lipinski rule [13], Caco-2, MDCK, total polar surface area, no. of rotatable bonds, and logBB, logPoct descriptors were identified. Madin-Darby canine kidney (MDCK) cell model and Caco-2 cell model are being suggested as a consistent in vitro model for the calculation of oral drug absorption. Blood–brain barrier (BBB) diffusion is critical in the pharmaceutical field since CNS-active compounds should essentially pass through it. It is presented as the quantitative ratio of stable-state of the concentration of compounds in the brain (C_brain_) and peripheral blood (C_blood_) [14]. For a molecule to be orally active it is essential to exhibit several Hydrogen bond donors less than 10, the number of Hydrogen bond acceptors less than 5 and predicted octanol/water partition coefficient should less than 5 for. Compounds with a total polar surface area of more than 140 Å squared be likely to be poor at cell membranes permeability. The standard value for QPlogBB should be within 2.0–1.2. The value of QPPMDCK and QPPCaco must be > 500 are considered to be great.

### Determination of DPPH scavenging activity

**Fig. 7:**
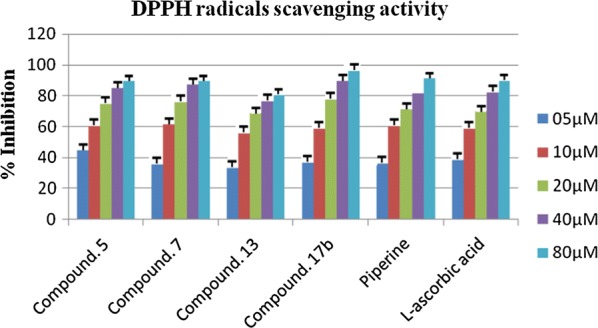
DPPH radical scavenging activity of most active compounds concerning reference l-ascorbic acid

Besides their inhibitory action on hMAO the antioxidant potential of synthesized compounds was investigated using 1,1-Diphenyl-2-picrylhydrazyl (DPPH) assay [15]. DPPH was utilized as a stable free radical because free radicals simulate reactive nitrogen and oxygen species distressing neurological structure. As indicated in Table 4, the compounds **5**, **17b**, and **7** showed very modest antioxidant activity with IC_50_ values as 5.553 ± 0.007 µM, 8.281 ± 0.001 µM, and 8.815 ± 0.019 µM, respectively. Whereas the reference compounds L-ascorbic acid and piperine exhibited IC_50_ values as 8.5.18 ± 0.009 µM and 9.814 ± 0.053 µM, respectively as shown in Fig. 7.

### Hydrogen peroxide scavenging activity

**Fig. 8:**
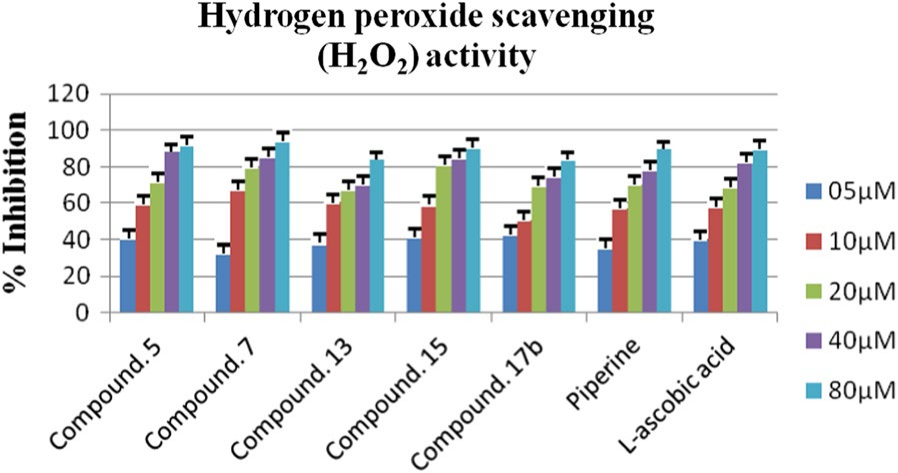
Hydrogen peroxide scavenging (H_2_O_2_) activity of most active compounds concerning reference l-ascorbic acid

The behavior for the H_2_O_2_ scavenging of all synthesized derivatives was evaluated and as depicted in Fig. 1, compounds **15**, **5**, **7** and **17b** exhibited concentration-dependent action for H_2_O_2_ scavenging with IC_50_ values 8.043 ± 0.005 µM, 8.279 ± 0.017 µM, 9.495 ± 0.045 and 10.09 ± 0.013 µM. Interestingly, compounds with good DPPH scavenging ability appeared as very modest H_2_O_2_ scavengers. However, the reference compounds l-ascorbic acid and piperine exhibited the IC_50_ values as 8.5.18 ± 0.009 µM, 11.33 ± 0.016 µM, respectively, as shown in Fig. 8.

## Discussion

Piperine based derivatives were synthesized by converting piperine to piperic acid by hydrolysis as shown in Scheme 1 and physicochemical data is given in Table 1. The structures of synthesized compounds were confirmed by spectroscopic techniques. Finally, synthesized compounds were investigated for the hMAO-A and hMAO-B inhibitory activities by using an extremely sensitive continuous fluorometric assay. It was observed that two compounds **7** and **17c** were found as significant hMAO-A inhibitors with IC_50_ values 15.38 ± 0.071 µM and 16.11 ± 0.091 µM, respectively. The docking scores of the compounds **7** and **17c** as − 9.72 and − 7.98 was also in agreement with the in vitro results [16, 17]. However, the reference compound clorgyline and piperine showed hMAO-A inhibitory activity with IC_50_ values of 18.74 ± 0.096 µM and 19.01 ± 0.031 µM, respectively. The reason for the more specific and potent hMAO-A inhibition could be due to the presence of bulky aromatic rings to the carbonyl amide position, this phenomenon can also be explained by the presence of large hydrophobic substrate cavity in hMAO-A which favorably allocated the bulky aromatic piperine based ligands within the active site. Moreover, umbelliferone hybrid compounds **13** with dock score − 6.87, also shown considerable hMAO-A activity with good selectivity. Which also supports the concept of bearing bulky moieties for better affinity towards hMAO-A. Moreover, a study of Mu and Leonard also predicted that the presence of small amine moieties on piperidine ring affords improved selective MAO-B inhibition in contrast to MAO-A [18, 19] (Table 2).

**Table 1:** Physicochemical properties of synthesized compounds

Sr. no	Chemical structure	R_f_ value	(%) Yield^a^	M.P
**5**		0.83	75.8	231–232
**7**		0.79	76.7	245–246
**9**		0.85	71,9	231–232
**11**		0.78	68.5	245–246
**13**		0.86	72.5	276–277
**15**		0.81	77.2	189–190
**17a**		0.83	73.8	210–211
**17b**		0.85	69.3	230–231
**17c**		0.79	71.9	192–193

^a^TLC mobile phase: hexane:toluene:ethyl acetate (1:1:1)

**Table 2:** MAO inhibitory activity of piperine derivatives

Sr. no	hMAO-A IC_50_, µM^a^	hMAO-B IC_50_, µM^a^	Docking score hMAO-A	Free energy of binding, kcal/mol (hMAO-A)	Docking score hMAO-B	Free energy of binding, kcal/mol (hMAO-B)	Selectivity index^b^
**5**	60.19 ± 0.087	14.19 ± 0.007	− 2.34	− 13.4	− 10.56	− 56.9	4.241
**7**	15.38 ± 0.071	52.20 ± 0.006	− 9.72	− 46.9	− 3.24	− 11.7	0.294
**9**	22.19 ± 0.07	71.01 ± 0.005	− 8.76	− 32.1	− 1.24	− 3.2	0.312
**11**	33.72 ± 0.071	20.59 ± 0.006	− 5.76	− 16.9	− 3.89	− 14.5	1.637
**13**	24.53 ± 0.002	65.99 ± 0.002	− 6.87	− 15.8	− 2.43	− 13.6	0.371
**15**	30.87 ± 0.005	12.15 ± 0.003	− 5.67	− 16.4	− 11.76	− 59.5	2.540
**17a**	32.33 ± 0.043	21.37 ± 0.007	− 4.84	− 12.9	− 8.65	− 33.2	1.512
**17b**	30.00 ± 0.005	17.90 ± 0.038	− 6.32	− 15.2	− 8.65	− 32.8	1.675
**17c**	16.11 ± 0.091	26.44 ± 0.063	− 7.98	− 16.2	− 5.43	− 17.3	0.609
Piperine	19.01 ± 0.031	17.57 ± 0.037	− 5.56	− 15.6	− 9.44	39.5	1.081
Clorgyline	18.74 ± 0.096	–	− 5.773	− 16.5	–	–	–
Harmine	23.12 ± 0.041	–	− 4.211	− 11.9	–	–	–
Pargyline	–	20.04 ± 0.095	–	–	− 6.061	− 15.3	–
Safinamide	–	24.23 ± 0.011	–	–	− 5.760	− 16.0	–

^a^Values related for the evaluated compound absorption which provide 50% MAO-A and MAO-B inhibition, action, and are the mean SEM; statistical significance: p < 0.05 against the equivalent IC_50_ values achieved against MAO-A and MAO-B, as identified through ANOVA/Dunnett’s test

^b^Selectivity index = IC_50_ (MAO-A)/IC_50_ (MAO-B)

For hMAO-B compounds **15** and **5** were found as considerable hMAO-B inhibitors along with good selectivity comparing with piperine with docking score − 11.76 and − 10.56, respectively. In compound **15** presence of electron-withdrawing group nitroso group on the naphthalenyl ring could be responsible for better binding (π–π) interactions and formation of the bridge within the active site of hMAO-B. Moreover, compound **5** with bromo group also indicated that the presence of electron-withdrawing groups on pharmacophore structure increases the MAO-B inhibitory activity. In contrast, another compound **17c** comprising esteric vanillin substitution at carbonyl amide position (IC_50_—17.90 ± 0.038 µM) was found as the considerable hMAO-B inhibitor. The cause for such divergence is not perceptible but bearing electron**-**withdrawing 4-formyl group on phenyl ring is expected to be among plausible explanations.

Another unexpected behavior for MAO inhibition was shown by compound **9**, which was found at least an MAO-B inhibitor. The reason for poor hMAO-B activity may be due to the presence of oxygen in the basic piperidine moiety attached through a carbonyl amide linkage in piperine. This trend of compound **9** could be explained by the outcomes of Al-Baghdadi and coworker’s study which indicated that the inclusion of oxygen in the ring decreases the MAO-B potential [10]. The kinetic study of the inhibition of compounds **7**, **15** against hMAO-A and hMAO-B was specified to be competitive.

Molecular docking was carried out for most active compounds for MAO-A and MAO-B inhibition. The compound **7** was positioned near to the vicinity of FAD and established hydrophobic interactions with Ile180, Tyr407, Ile335, Cys 323, Leu97, Val 93, Ala 110 residues via aromatic part of the ligand such ligand interactions were also reported by Hubalek and coworkers [20]. Moreover, within the active site of hMAO-A, polar Thr336, Gln 215 and Asn 181 were embedded in the front of *N*-(naphthalen-1-yl) moiety as of both sides, although free rotation of this group would promise the absence of any adverse interactions with these amino acids. However, **17c** showed the formation of a π–π stacking bond with Phe108 and phenolic ring, contributed notable binding within the active site along with docking score of − 5.43. The double bonds in conjugated form were entrenched in a huge hydrophobic compartment created by Val 210, Ala 111, Leu 97, Cys 323 residues. Additionally, polar residues such as Ser209 and Thr211 were appeared to be approached by the methylenedioxyphenyl ring of **17c**. Another residue Gln215 seemed closed to ester linkage of **17c**.

In case of MAO-B, the hydrophobic interactions were formed by Cys172, Leu 171, Ile198, Phe168, Pro104, Trp119 and a ‘gatekeeper’ residue Ile199 with compound **15** as reported by Binda and coworkers [21]. Methylenedioxyphenyl moiety of **15** was emerged out along with side chain through polar residues such as Thr314, Thr201, Ser200, Asn203, and Thr202. The methylenedioxyphenyl unit of **5** was surrounded by polar residues such as Thr202 and Asn203. Moreover, the 4-bromophenyl was the ring of **5** was embedded into the ‘aromatic cage’ enclosed by Phe99, Phe103, Trp119, and Phe168 residues.

Docking studies confirmed that the important scaffold which is compulsory for the inhibitory activity. Consequently, slight alterations of substituent dimension in piperine lead compound were barely accepted. Such behavior could be also defensible through the in-depth structural data that PDB granted regarding the hMAO-A and hMAO-B active site: the dynamic site of hMAO-A composes of a single huge hydrophobic cavity that acclimatizes itself for the bulkiest aromatic ligands constructively rather than two petite hydrophobic pockets of hMAO-B. Therefore, the absence of hMAO selectivity and activity were found highly affected via steric interference of the piperine lead substitution. In the case of ADMET study, the score values by QikProp revealed that the majority of ADME properties have a positive impact on compounds to be a suitable candidate for further development.

In the case of DPPH scavenging activity the compounds **5**, **17b**, and **7** showed very modest antioxidant activity with IC_50_ values as 5.553 ± 0.007 µM, 8.281 ± 0.001 µM and, 8.815 ± 0.019 µM, respectively. Whereas the reference compounds L-ascorbic acid and piperine exhibited IC_50_ values as 8.5.18 ± 0.009 µM and 9.814 ± 0.053 µM, respectively. Notice that the compounds with good antioxidant potential also appeared as very modest anti-MAO agents. The DPPH scavenging action based on the primary method and the capacity of the compound to hydrogen atom transfer [22]. So this may be the reason for the potential antioxidant activity of the compounds **5**, **17b**, and **7** due to the presence of hydrogen as 2,4-dienamide in **5** and **7,** 2-methoxyphenyl in **17b**. Furthermore, to be antioxidant these compounds should have the lesser bond dissociation energies of N–H and O–H in their structures. Moreover, compounds **17b**, **5**, and **7** showed good H_2_O_2_ scavenging activity. Fundamentally the H_2_O_2_-scavenging action is mainly reliant upon the hydrogen-losing capacity of synthesized compounds and the constancy of phenoxyl radicals produced subsequently to the dehydrogenation. Therefore it can be concluded that the most active H_2_O_2_ scavenging compounds are capable to donate hydrogen via 2,4-dienamide in **5** and **7** and **7,** 2-methoxyphenyl in **17b**.

## Experimental

### Materials and methods

Unless otherwise noted, the analytical grade chemicals required for synthesis and antioxidant activity procured from Sigma Aldrich, Merck, Hi-media Laboratories. The analytical assessment of the synthesized derivatives for hMAO inhibition was measured by their effects on the generation of hydrogen peroxide (H_2_O_2_) by p-tyramine (a general substrate for both MAO isoforms), utilizing the Amplex Red MAO assay kit (Sigma USA) and microsomal MAO enzymes extracted by insect cells (BTI-TN-5B14) expressed on recombinant baculovirus containing cDNA probes for MAO isoforms (Sigma-Aldrich USA) were used as source for the two microsomal MAO isoforms. The progress of the reaction was cheeked through thin layer chromatography TLC executed on 0.25 mm pre-coated plates with silica gel procured from Merck, and the spots were envisaged in iodine chamber and UV cabinet, in mobile media TLC-hexane:toluene:ethyl acetate (1:1:1). Melting points were recorded on Sonar melting point apparatus in open capillary tubes. The nuclear magnetic resonance (NMR) spectra 1H NMR and ^13^C NMR spectra were determined in DMSO and deuterated CDCl_3_ respectively on a spectrometer (Model: Bruker Avance II 400 NMR). Coupling constants J are in hertz (Hz) (Additional file 1). Spectral data for infrared (IR) was confirmed on Perkin Elmer FTIR spectrophotometer by using KBr pellets technique.

### General procedure for the preparation of piperic acid [23]

In a 1-l round-bottom flask was placed piperine (6 g), ethanol (500 ml) and KOH as the catalyst were refluxed at 80 °C for 3 days. After the completion of the hydrolysis of piperine, the reaction mixture was then cooled and the precipitates were filtered off and dispersed in acidified warm water (HCl to pH 1). Precipitates as yellow color were attained by filtration, washed down by chilled water and recrystallized in ethanol to produce piperic acid crystals.

### General procedure for the synthesis of piperic acid chloride

Piperic acid (20 mmol, 2.46 g) was reflux with stirring 80 ℃ for 2–4 h, treated with thionyl chloride (10–15 ml) in the presence of few drops of pyridine as a catalyst. The residual amount of thionyl chloride was removed through distillation. The purity of piperic acid chloride was appropriate to use the product directly for the following synthesis.

### General procedure for the synthesis of amides [24]

Compound 5 and compound 7 and 9 were synthesized by dropwise addition of p-bromo aniline, naphthylamine and morpholine (0.1 mol) to a solution of piperic acid chloride (0.1 mol) into ether 50 ml ether kept at 0–05 °C temperature. This reaction was carried out by stirring for 30 min maintaining the cold condition as the reaction is exothermic and finally, the precipitated amides were separated by filtration. The crude precipitated compounds were washed with 5% HCl, 4% Na_2_HCO_3_ in water to eliminate impurities and the consequential amides were recrystallized by methanol.

### General procedure for the synthesis of esters

The titled esters derived from piperic acid chloride (0.05 mol) were prepared by refluxing different aromatic alcohols in ether (50 ml) at 80 °C for 6–8 h. The reaction combination was refluxed in a water bath in anticipation of the formation of hydrogen chloride was completed; the reaction was observed by TLC as showing a single spot. Finally, the ether deposit was divided and upon evaporation afforded the crude esters; these were then recrystallized by methanol.

### Human MAO-A and MAO-B inhibitory activity

To evaluate the MAO inhibitory potential of piperine based derivatives, the assays were conducted using a similar procedure as previously described Anderson and Chimenti with some modifications [25, 26]. Human MAO-A and -B recombinant enzymes (Sigma-Aldrich) 5 mg ml^−1^ were pre-aliquoted and placed at − 70 °C. The interactions of the titled compounds with hMAO isoforms were evaluated through a fluorimetric method based on the H_2_O_2_ formation rendered by MAO isoforms was identified by utilizing the non-fluorescent, extremely susceptible and stable probe Amplex^®^-Red reagent (10-acetyl-3,7-dihydroxyphenoxazine), which reacts with H_2_O_2_ in the existence of horseradish peroxidase to generate resorufin which is a fluorescent component. Concisely, the reaction mixture with 0.1 ml of sodium phosphate buffer (0.05 M, pH 7.4) having different ranges of concentration of the piperine based derivatives and standard drugs and appropriate quantity of hMAO-A or hMAO-B recombinant isoforms necessary for oxidation of (in control group) para-tyramine (165 pmol) min^−1^ (hMAO-A: 1.1 μg protein; specific activity: 22 nmol of para-tyramine oxidized to *p*-hydroxyphenylacetaldehyde per min, per mg protein; MAO-B: 7.5 μg; specific activity: 150 nmol of para-tyramine transformed per min per mg protein) were incubated for 15 min at 37 °C in semi-micro fluorometry cuvettes and placed into the dark fluorimetric chamber. By the incubation time, the reaction was initiated via the addition of 200 μM Amplex Red reagent, 1 mM *p*-tyramine (general substrate for both MAO isoforms) and 1 U ml^−1^ horseradish peroxidase (HRP). Finally, the generation of H_2_O_2_ and thus, of resorufin was estimated at 37 °C in fluorescence multi-well plate scanner (λ_excitatio_ = 530 nm, λ_emission_ = 585 nm) for 15 min time, a phase wherein fluorescence amplified linearly as of the starting. The probable interaction of the piperine based derivatives with Amplex Red reagent was detected through the addition of the compounds to solutions including the only Amplex Red reagent in a sodium phosphate buffer. The calculation of specific fluorescence emission (used to obtain the final results) was carried out by subtraction of background action that was identified by the cuvettes comprising all reagents except the MAO isoforms, which were changed by a sodium phosphate buffer solution. The preparation of stock solutions and all reagents was done newly each day in deionized water and kept at − 20 °C. Data were processed in Microsoft Excel, to calculate IC_50_ values.

### Enzyme kinetics

The enzymatic kinetic factors maximum reaction rate (V_max_) and Michaelis–Menten constant (K_m_) were computed by GraphPad Prism Software 6.02 (San Diego, CA, USA). The IC_50_ values were determined by nonlinear regression best fit model of normalized response with variable slope. Lineweaver–Burk plot was obtained from Michaelis–Menten data. The Michaelis–Menten constant (Km) is the concentration of inhibitor compound in the presence or absence of inhibitor, respectively. Relative selectivity (RS) folds were defined by the ratio of *h*MAO-A IC_50_ to *h*MAO-B IC_50_. The inhibition mechanism was identified by the double reciprocal plot (Lineweaver–Burk curves) by plotting the inverse of the substrate concentration (1/S) as a function against the inverse of initial velocity (1/V).

### Preparation of ligands

Theoretical 3D models of all compounds were built by Chem Draw in MDL MOL form and afterward, LigPrep (Schrödinger) was used to optimize the structures by and assigning them correct protonation positions and torsions were optimized for ligands [27]. The generation of around 32 stereochemical structures per ligand at pH 7.0 ± 2.0 states using Ionizer, further the ligands were desalted, tautomerized and optimized trough generating 3D low energy structures computed via OPLS 2005 force field. The chiralities of ligands were retained as such and minimization of energy was done by MMFF force field within Glide (Schrödinger).

### Preparation of proteins

The protein crystal structures for hMAO-A and hMAO-B in the high resolution were retrieved from the Protein Data Bank with accession codes 2Z5X [28] and 2V5Z [29], respectively. Before proceeding the docking experiments the co-crystallized inhibitors, harmine for 2Z5X and safinamide for 2V5Z and structural water molecules were detached from hMAO-A and hMAO-B proteins. The definite binding pockets were specified via 1000 A3 regular grid box positioned towards N5 atom of cofactor FAD. Finally, the charge and protonation states were assigned and energy minimization was carried out by the OPLS2005 force field.

### Docking methodology

The computational docking experiments were performed by adopting XP precision mode using a flexible algorithm within Glide [30]. The box size was produced repeatedly for selection and creation of centroid of the active site residues and the intact protein receptor was subjected for energy minimization along with RMSD value of 0.18 Å. Before the docking experiments, the side chains based on B-factor were separated along with co-crystalline ligands (inhibitors) present at Van der Waals degree of 0.69 and 0.49, respectively; around 20 poses were generated for each ligand. The residues were refined and calculation, optimization, and minimization of the leading side chain were performed within 5.2 Å of ligand poses. The abovementioned methods revealed the exact position of conformation and ligand structure for the induced fit method. Consequently, the redocking was done in Glide XP mode in which the given docking score for each ligand.

### In silico ADMET study

The ADMET study of the entitled compounds was accessed by the use of QikProp module of the Schrödinger along which provided the in silico data about the oral absorption and certain toxic parameters Table 3. The Pfizer’s rule of five was followed for the oral absorption that regulates the molecular descriptors such as topological molecular weight (MW) below 500 Daltons, polar surface area ≤ 140 Å (TPSA), maximum 10 hydrogen bond acceptor (HBA), LogP (not surpass 5) and maximum 5 hydrogen bond donor (HBD) should be within the acceptable range for drug-like character. Number of rotatable bonds should be ≤ 10 according to Lipinski rule. Because the maximum number of rotatable bonds increases the ligand flexibility moreover, it provides the better molecular interactions within the binding site.

**Table 3:** *In silico* ADMET profile of Piperine derivatives

Sr. no	Mol. wt	TPSA	No. of rotatable bonds	DonorHB	AccptHB	QPlogPo/w	QPlogBB	QPPMDCK	QPPCaco
**5**	372.21	47.57	4	1	4	4.34	0.134	4525.13	3140.96
**7**	343.38	47.57	4	1	4	4.549	0.429	1318.41	2476.54
**9**	277.32	48.01	3	0	6	2.15	0.118	2146.11	3887.00
**11**	356.46	44.77	6	0	3	5.445	0.229	2470.55	4427.72
**13**	362.33	74.98	5	0	6	2.955	0.939	408.022	836.747
**15**	373.36	74.21	6	0	5	3.846	0.945	484.356	980.622
**17a**	350.15	44.77	6	0	4	2.78	1.088	392.825	807.873
**17b**	352.34	71.08	8	0	7	2.18	1.874	234.985	708.548
**17c**	364.39	54.01	7	0	5	4.949	0.377	2442.11	4380.54

### Determination of DPPH radicals scavenging activity

The DPPH method is one of the most proficient techniques to assess the radical-scavenging action by a chain-breaking mechanism. The antiradical potential of the piperine based compounds was estimated alongside 2,2-diphenyl-2-picrylhydrazyl hydrate (DPPH) a stable free radical, was evaluated spectrophotometrically. DPPH is stable free radical on room temperature, however on acceptance of a hydrogen/electron radical it turns into a stable diamagnetic molecule. The scavenging activity of DPPH radical is assessed by the reduction in its optical density at 5l7 nm, rendered by antioxidants [31]. This phenomenal feature of DPPH is due to the reaction among antioxidant compounds and radicals, leads to the scavenging of the free radical via donated hydrogen. It manifested visually as the color bleaches from purple to yellow. Therefore, DPPH is generally utilized as a substrate to estimate the ant oxidative potential. Different concentrations of around fifty milliliters (25, 50, 75, and 100) µg ml^−1^ of the titled derivatives were suspended in methanol and mixed with 5 ml of a 0.004% methanol solution of DPPH. Subsequently the all dilutions were subjected to incubation for 30 min at 37 °C temperature; finally, at 517 nm the absorbance was measured against a blank. Ascorbic acid was utilized as a reference while and all the experiments were performed in triplicate. The calculated IC_50_ values are depicted in Table 4. DPPH free radical scavenging of compounds was calculated as scavenging activity for DPPH (%) = [(Ac − At)/Ac] × 100 Where Ac is the absorbance of the control reaction At is the absorbance of the test compound. The absorbance was measured with UV/Vis Epoch ELISA reader at 517 nm, and antioxidant activity was measured as a decrease in absorbance of DPPH·.

**Table 4:** DPPH radicals scavenging activity of piperine derivatives

Sr. no	IC_50_ µM^a^	Sr. no	IC_50_ µM^a^
**5**	5.553 ± 0.007	**15**	11.03 ± 0.066
**7**	8.815 ± 0.019	**17a**	17.15 ± 0.026
**9**	12.35 ± 0.021	**17b**	8.281 ± 0.001
**11**	14.15 ± 0.011	**17c**	16.52 ± 0.012
**13**	10.70 ± 0.035	Piperine	9.814 ± 0.053
l-Ascorbic acid	8.5.18 ± 0.009		

^a^Value are expressed as mean ± SEM, n = 3

### Hydrogen peroxide scavenging (H_2_O_2_) assay

H_2_O_2_ is quickly decomposed into oxygen and water and this could generate hydroxyl radicals (OH) that may commence DNA damage in the body and lipid peroxidation. The hydrogen peroxide inhibitory activity of newly synthesized compounds was assessed by the technique illustrated by Nakano and coworkers with some modifications [32, 33]. In phosphate buffer (40 mM pH 7.4) the solution of hydrogen peroxide (40 mM) was prepared and different concentrations of titled compounds (05–80 μg ml^−1^) were added to H_2_O_2_ solution (2 ml). The concentration of hydrogen peroxide is determined by absorption at 230 nm using a spectrophotometer after 10 min of incubation. Blank reading was noted of phosphate buffer without H_2_O_2_. Results of antioxidant potential are shown in Table 3. The percentage of hydrogen peroxide inhibition was anticipated by the formula [(A_b_ − A_t_)/A_0_] × 100, where A_b_ is the absorbance of the control and A_t_ is the absorbance of compounds/standard taken as l-ascorbic acid (05–80 μg ml^−1^). UV/Vis Epoch ELISA reader was used to measure as a decrease in absorbance in the 96 wells (Table 5).

**Table 5:** Hydrogen peroxide scavenging (H_2_O_2_) activity piperine derivatives

Sr. no	IC_50_ µM^a^	Sr. no	IC_50_ µM^a^
5	8.279 ± 0.017	**15**	8.043 ± 0.005
**7**	9.495 ± 0.045	**17a**	23.76 ± 0.021
**9**	13.98 ± 0.010	**17b**	10.09 ± 0.013
**11**	17.57 ± 0.091	**17c**	15.71 ± 0.014
**13**	11.35 ± 0.004	Piperine	11.33 ± 0.016
l-Ascorbic acid	8.5.18 ± 0.009		

^a^Value are expressed as mean ± SEM, n = 3

## Conclusion

Together with all these findings, we developed a structural rationale for the hMAO-A and hMAO-B specificity of this innovative set of natural-based piperine inhibitors. Such outcomes enhanced our confidence in our project and inspired us to carry on our study to design more persuasive and selective inhibitors. Along with potent antioxidant activity, these compounds were corroborated as very modest lead compounds for the future pre-clinical studies. Despite their interesting therapeutic potential, they can be explored as the unique chemical template for the successive development and design of new drugs (selective and potent inhibitors of hMAO-A or hMAO-B) with improved pharmacological action and efficient against the risk factors of neuropsychological disorders and neurodegenerative diseases (e.g., Alzheimer’s disease), respectively.

## Supplementary information

**Additional file 1.** Spectral data of synthesised compounds.

## Data Availability

The datasets used and/or analyzed during the current study are available from the corresponding author on reasonable request.

## Abbreviations

AccptHB: Number of Hydrogen bond acceptors
DonorHB: Number of Hydrogen bond donors
ELISA: Enzyme-linked immunosorbent assay
FAD: Flavin adenine dinucleotide
MMFF: Merck Molecular Force Field
OPLS: Optimized Potentials for Liquid Simulations
QPlogBB: Calculated brain/blood partition coefficient
QPlogPo/w: Calculated octanol/water partition coefficient
QPPCaco: Calculated perceptible Caco-2 cell permeability in nm/sec
QPPMDCK: Calculated perceptible MDCK cell permeability in nm/sec
RMSD: Root-mean-square deviation
TLC: Thin layer chromatography
TPSA: Total polar surface area
