# Supplementary material for: Natural based piperine derivatives as potent monoamine oxidase inhibitors: an in silico ADMET analysis and molecular docking studies
Source: BMC Chem. 2020 Feb 17;14(1):12. doi: 10.1186/s13065-020-0661-0 (PMC7027018; doi:10.1186/s13065-020-0661-0)

Additional File 1 Spectral Data of synthesized compounds

Natural based piperine derivatives as potent monoamine oxidase inhibitors: an in silico ADMET analysis and molecular docking studies

Priyanka Dhiman[1], Neelam Malik[1] and Anurag Khatkar[2]

Series (IV) compound 5 IR

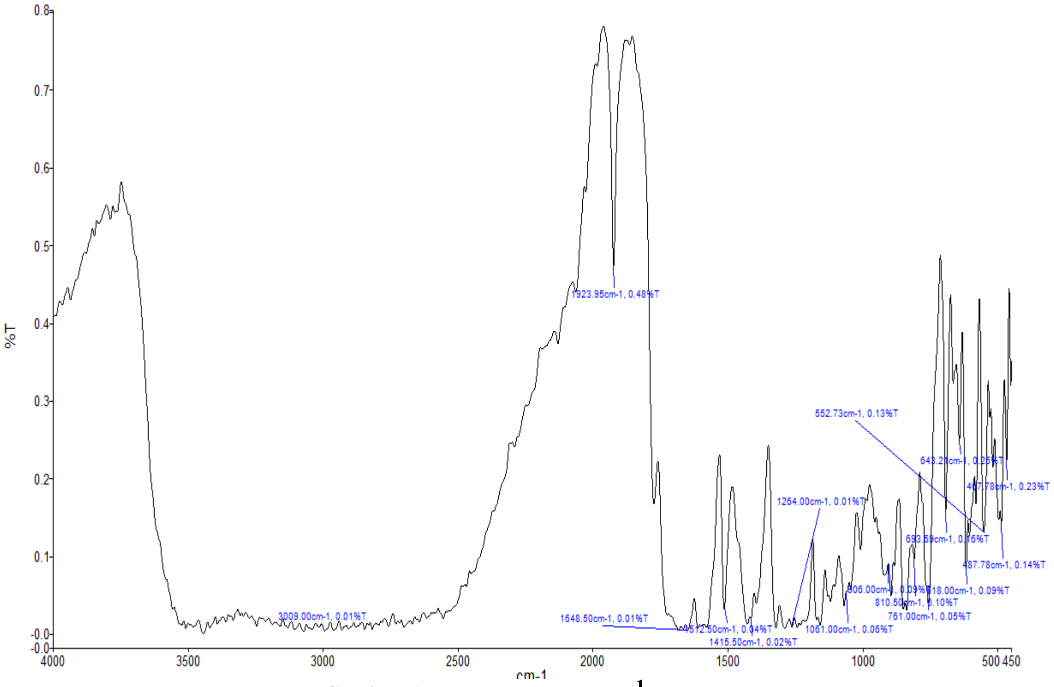

Series (IV) compound 5 ^{1}H-NMR

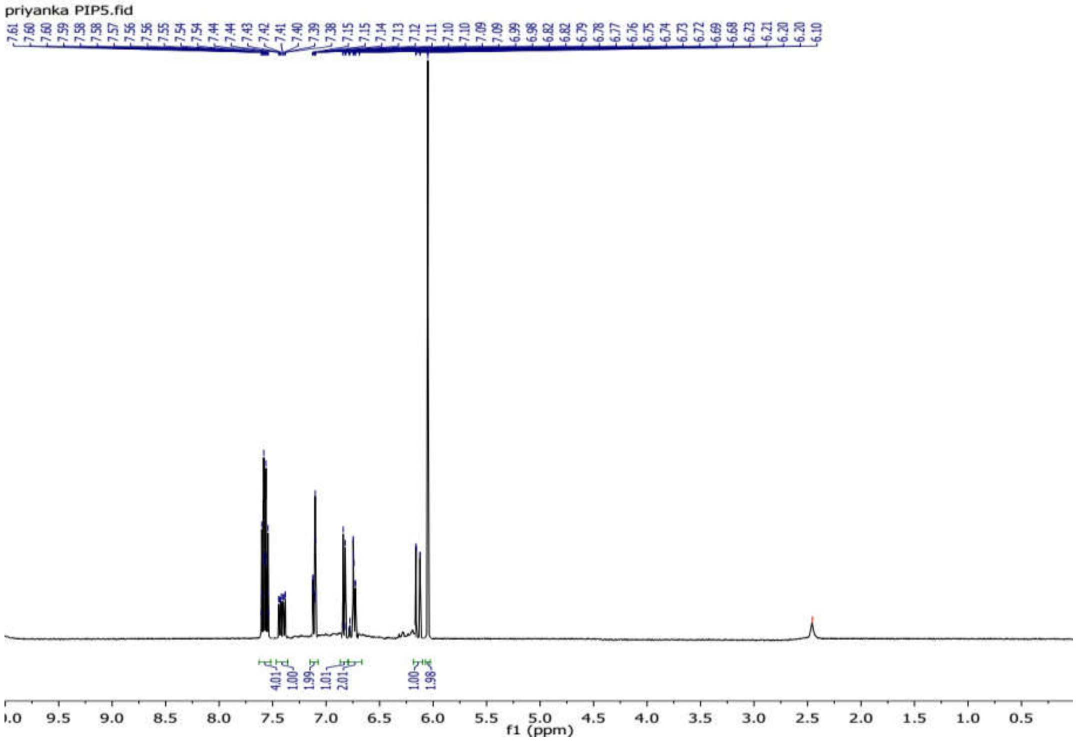

Series (IV) compound 5 ^{13}C-NMR

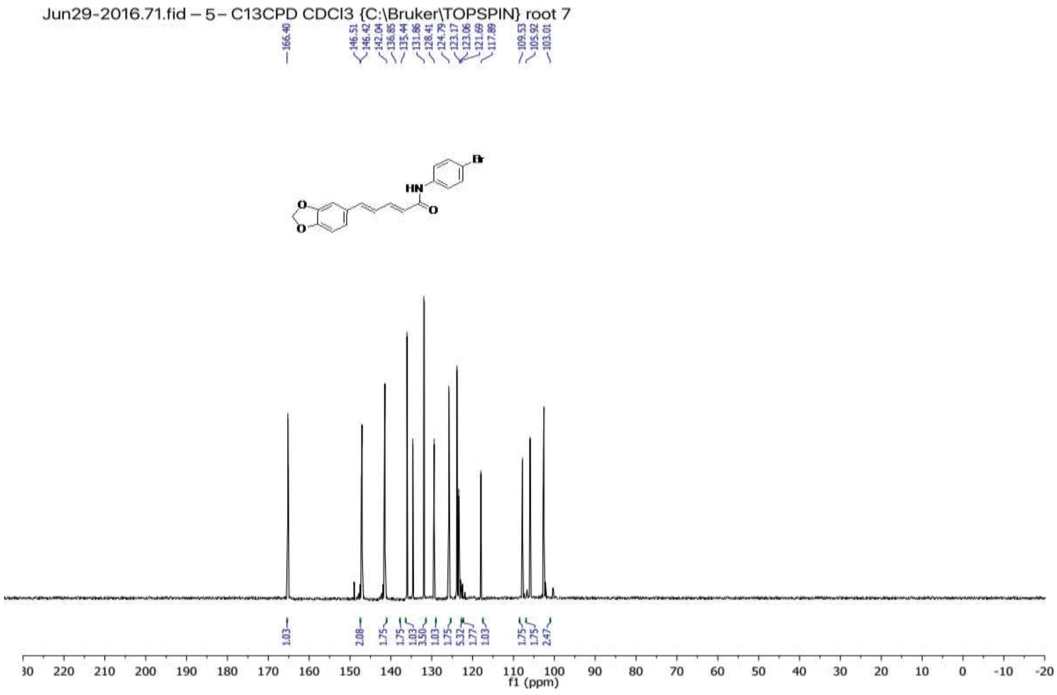

Series (IV) compound 7 IR

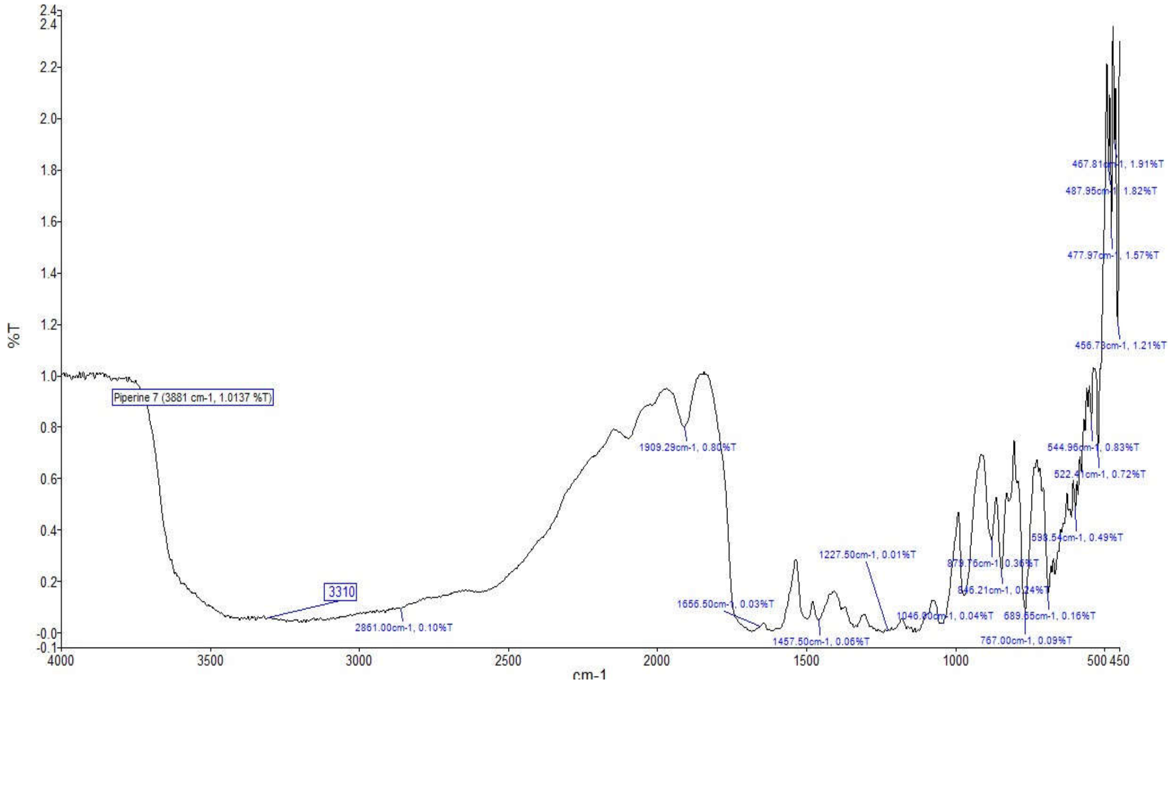

Series (IV) compound 7 ^{1}H-NMR

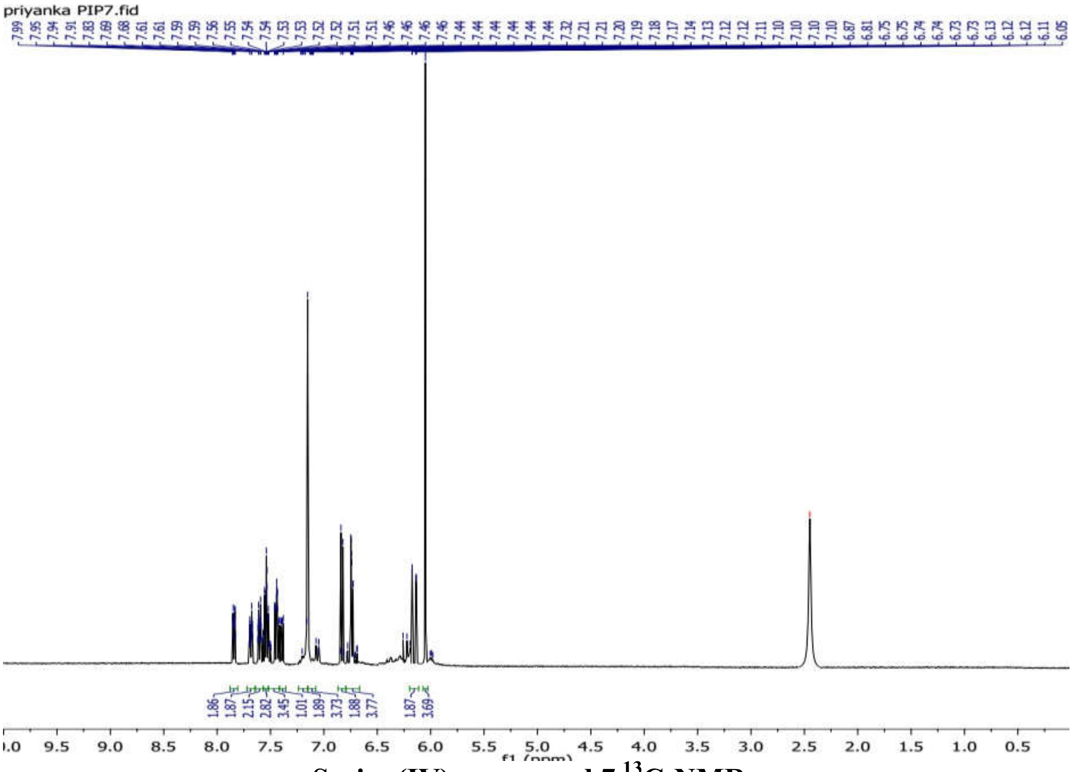

Series (IV) compound 7 ^{13}C-NMR

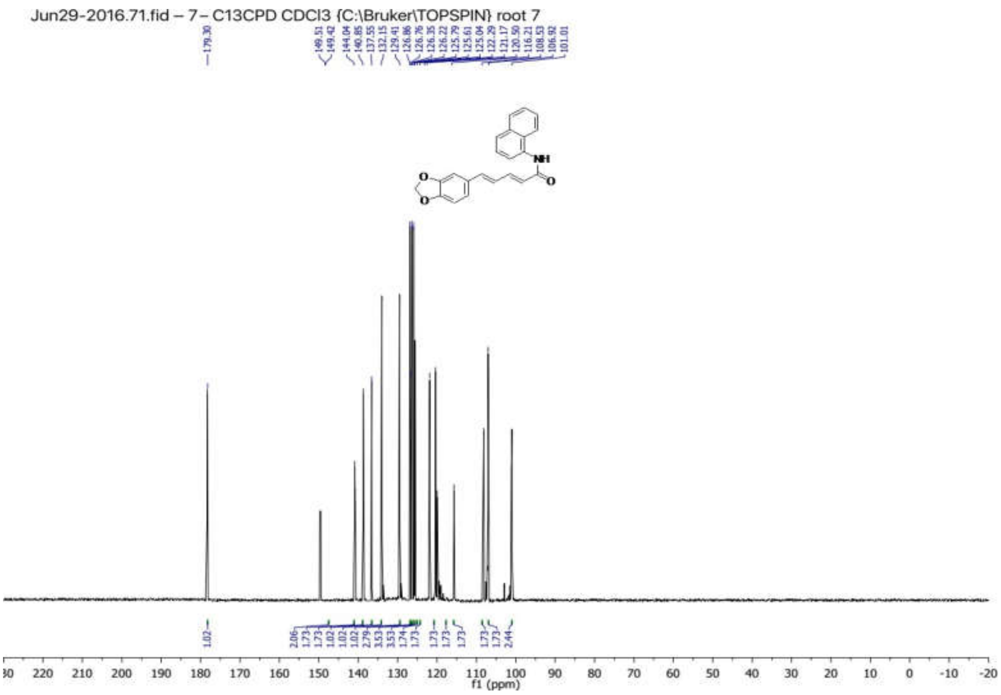

Series (IV) compound 9 IR

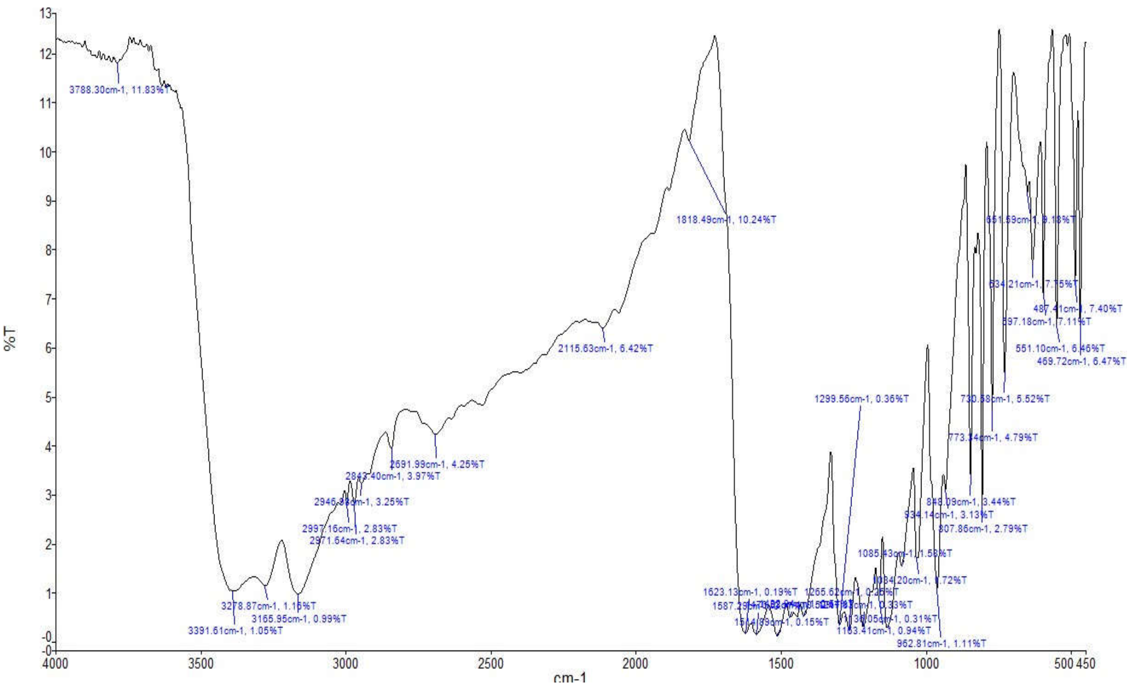

Series (IV) compound 9 ^{1}H-NMR

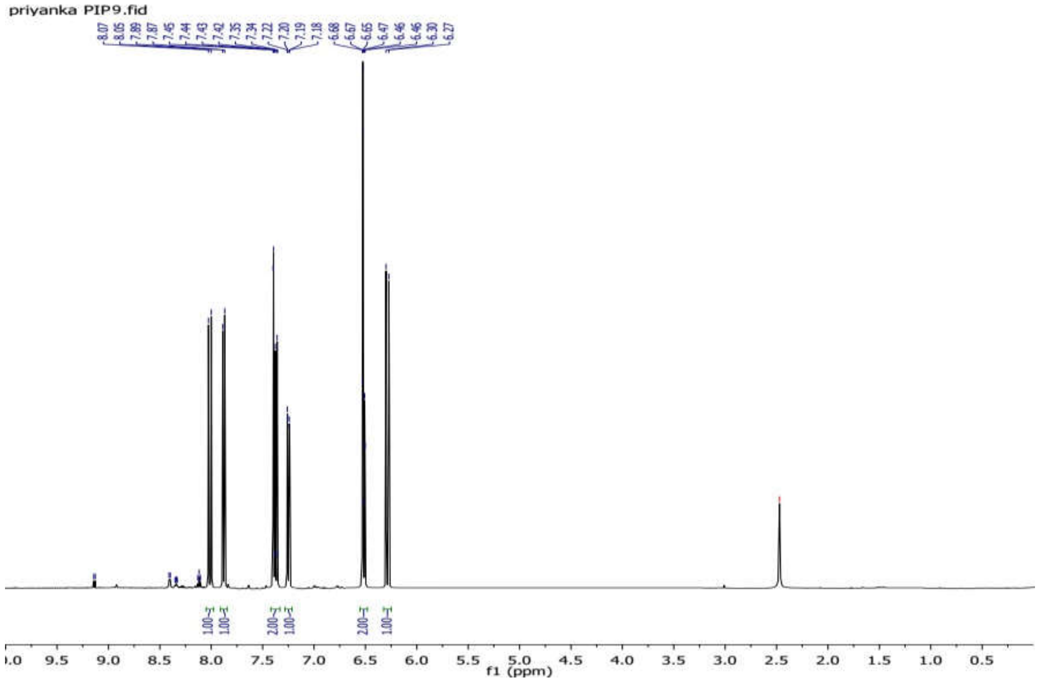

Series (IV) compound 9 ^{13}C-NMR

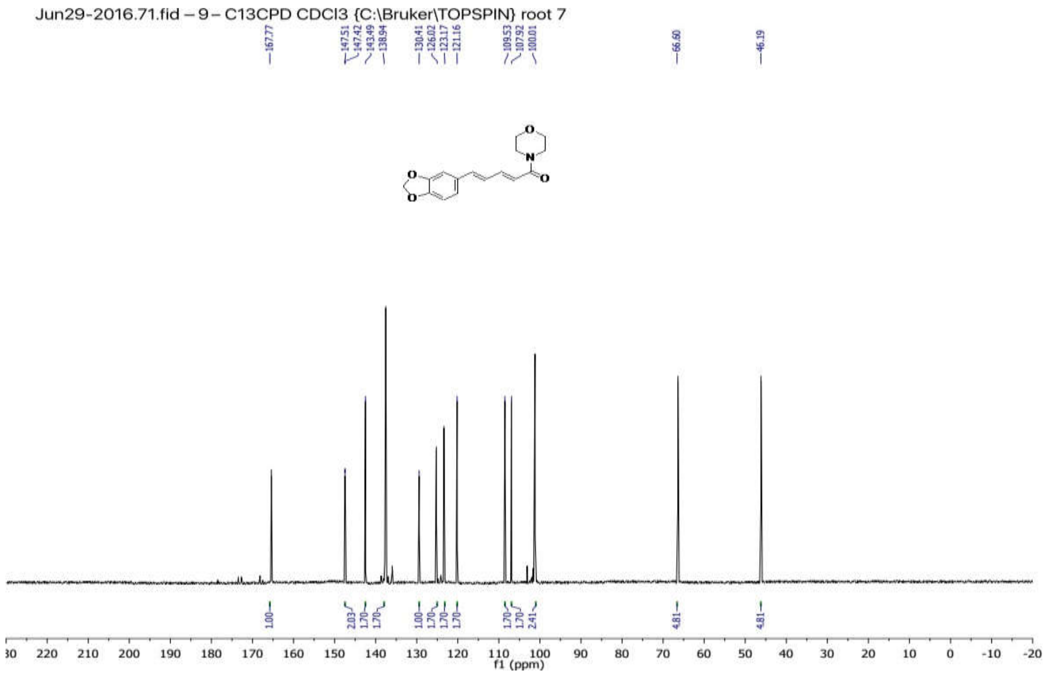

Series (IV) compound 11 IR

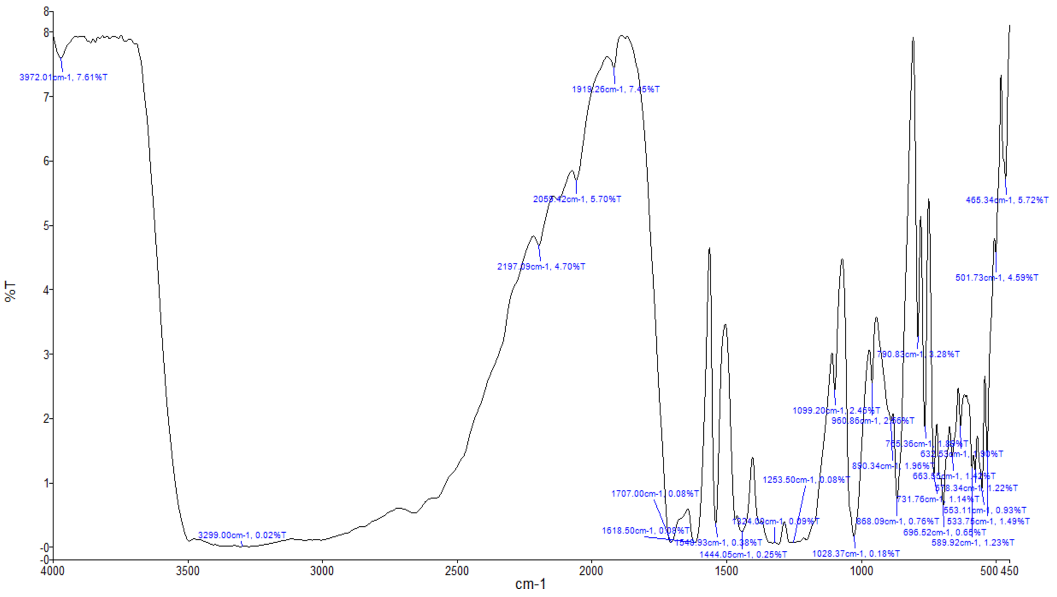

Series (IV) compound 11 ^{1}H-NMR

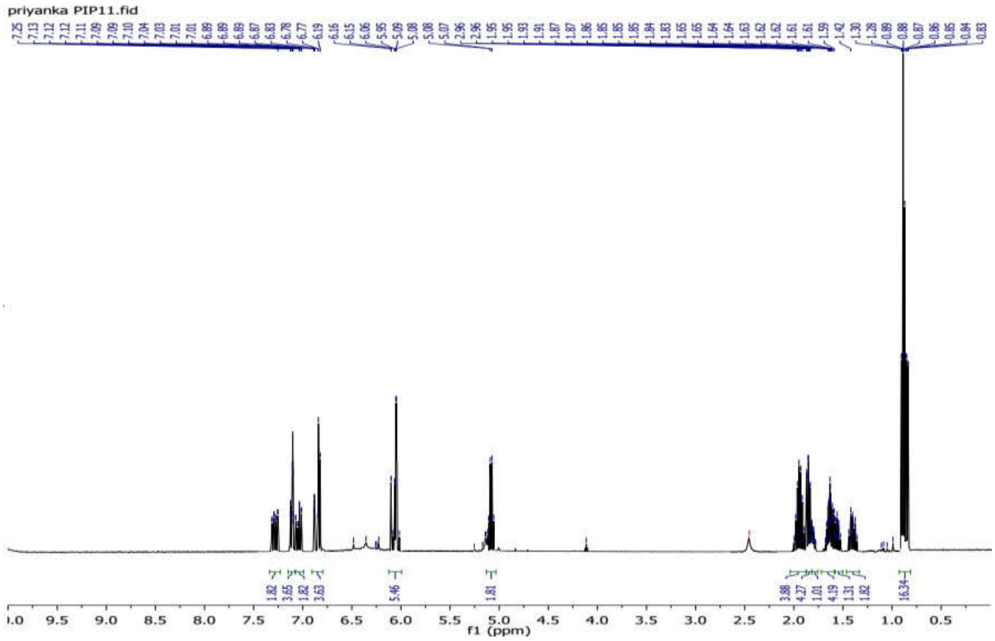

Series (IV) compound 11 ^{13}C-NMR

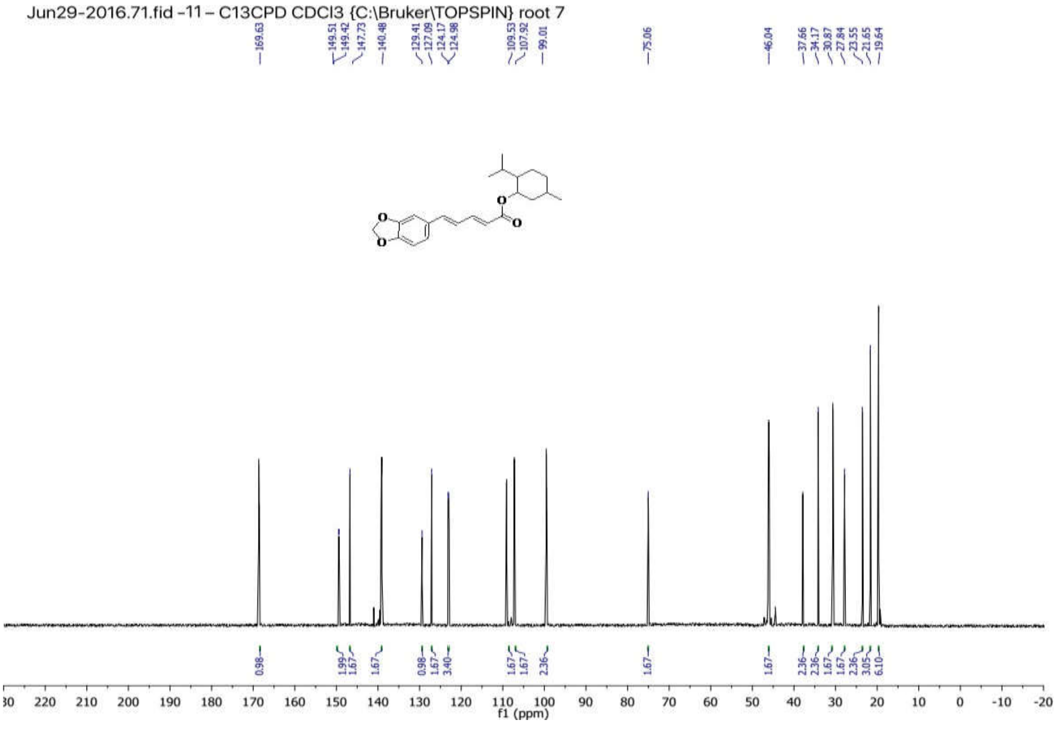

Series (IV) compound 13 IR

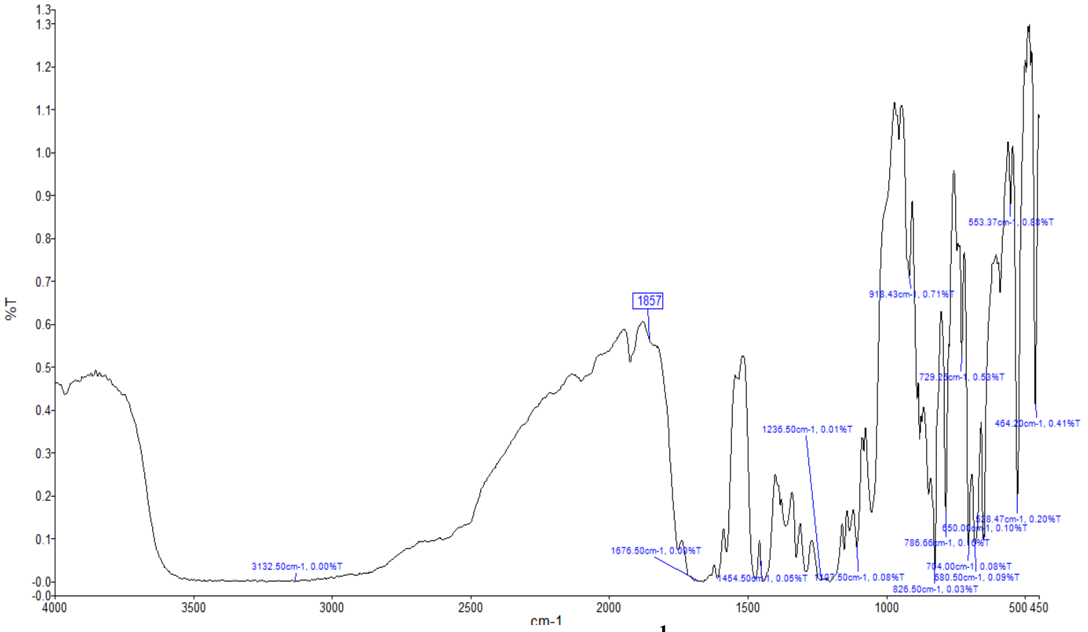

Series (IV) compound 13 ¹H-NMR

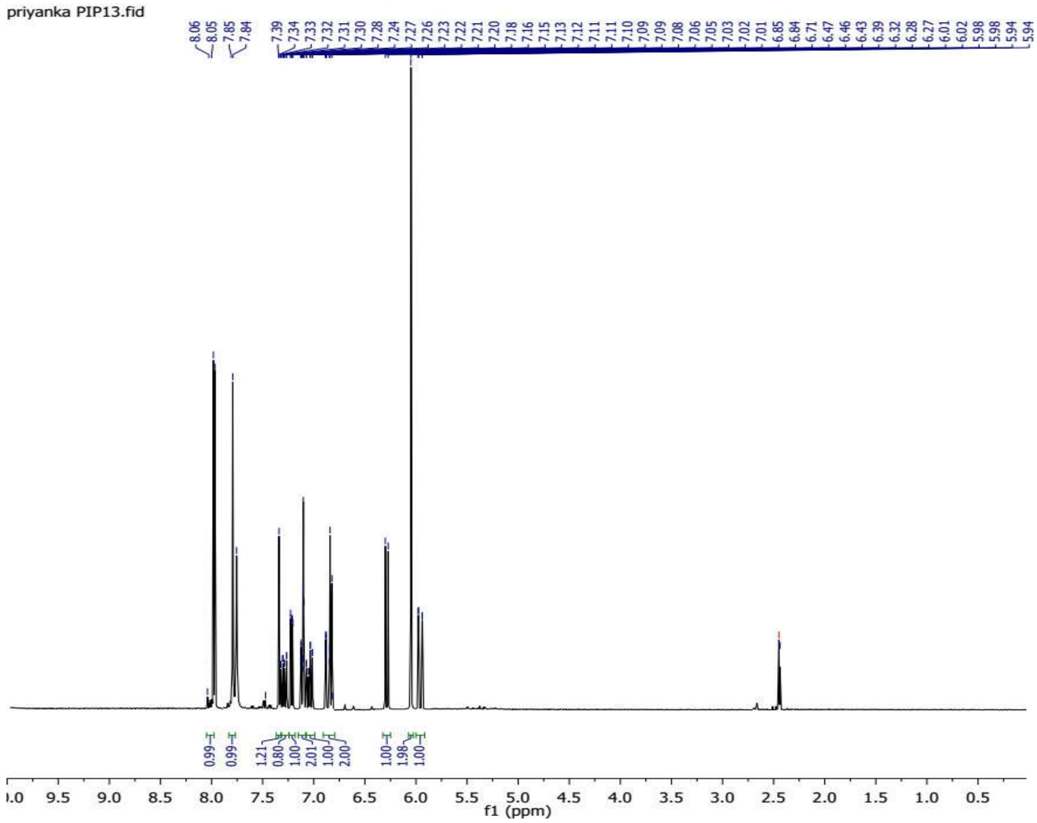

Series (IV) compound 13 ^{13}C-NMR

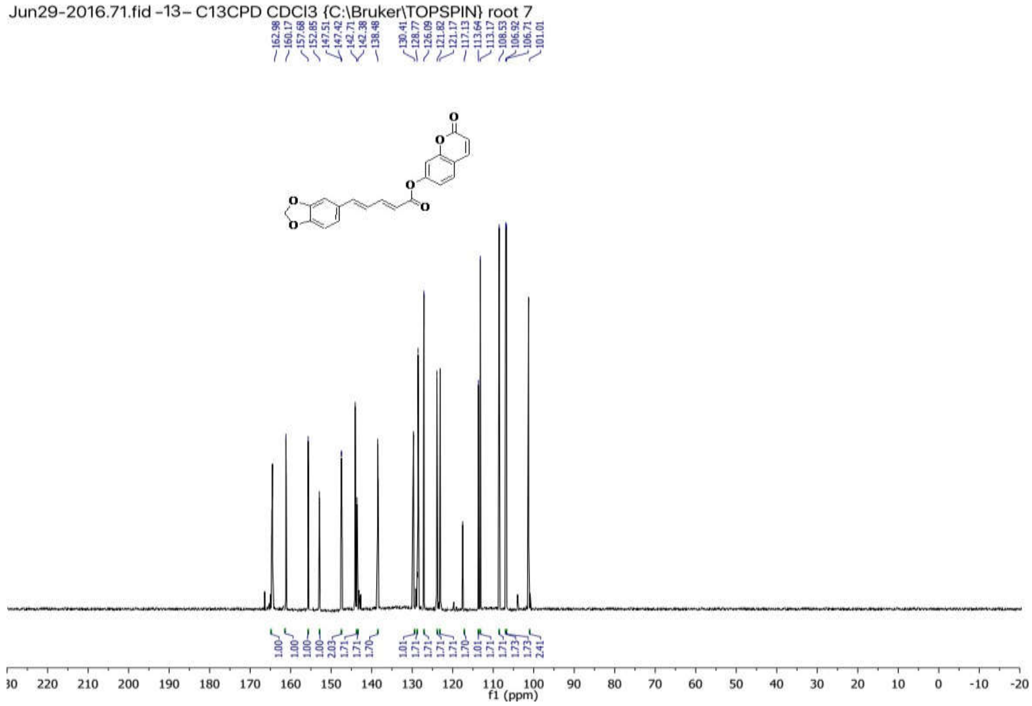

Series (IV) compound 15 IR

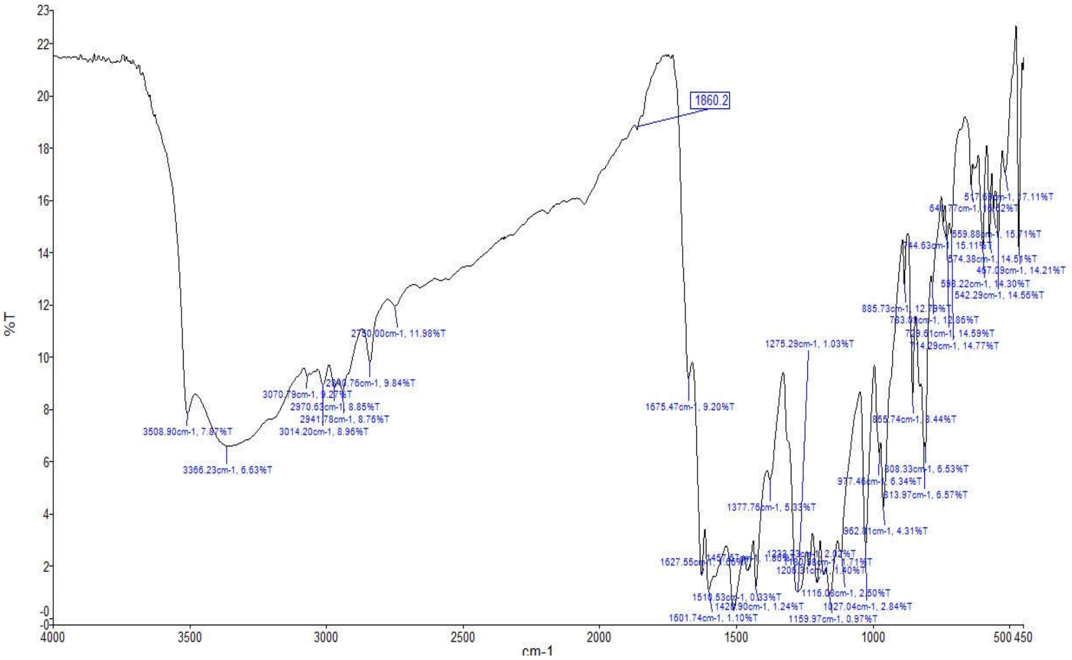

Series (IV) compound 15 ^{1}H-NMR

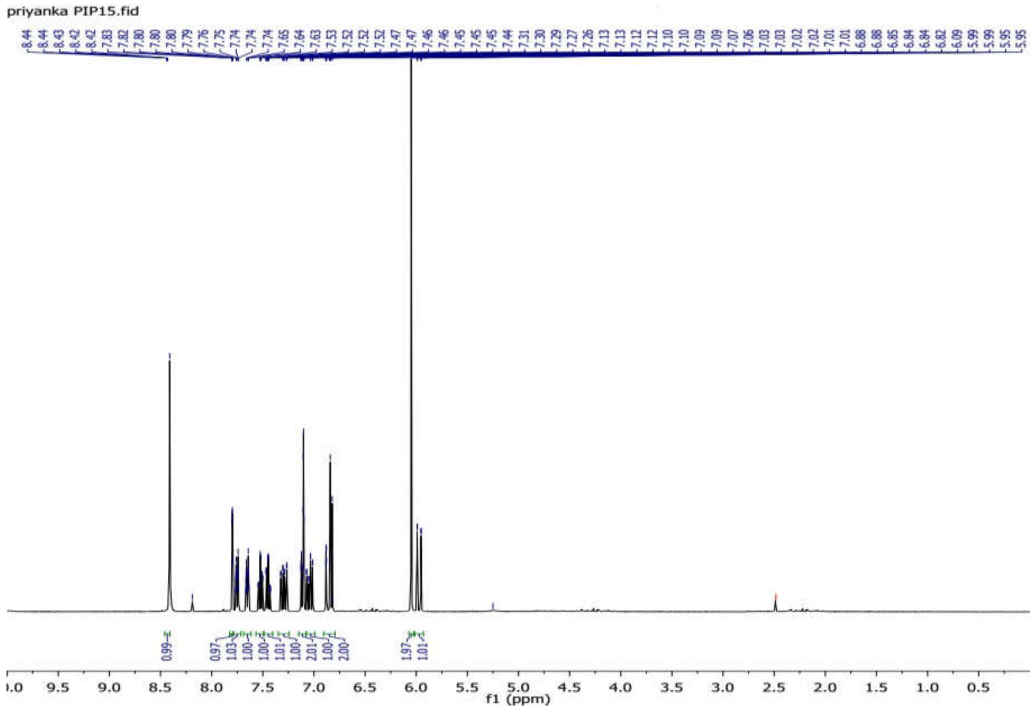

Series (IV) compound 15 ^{13}C-NMR

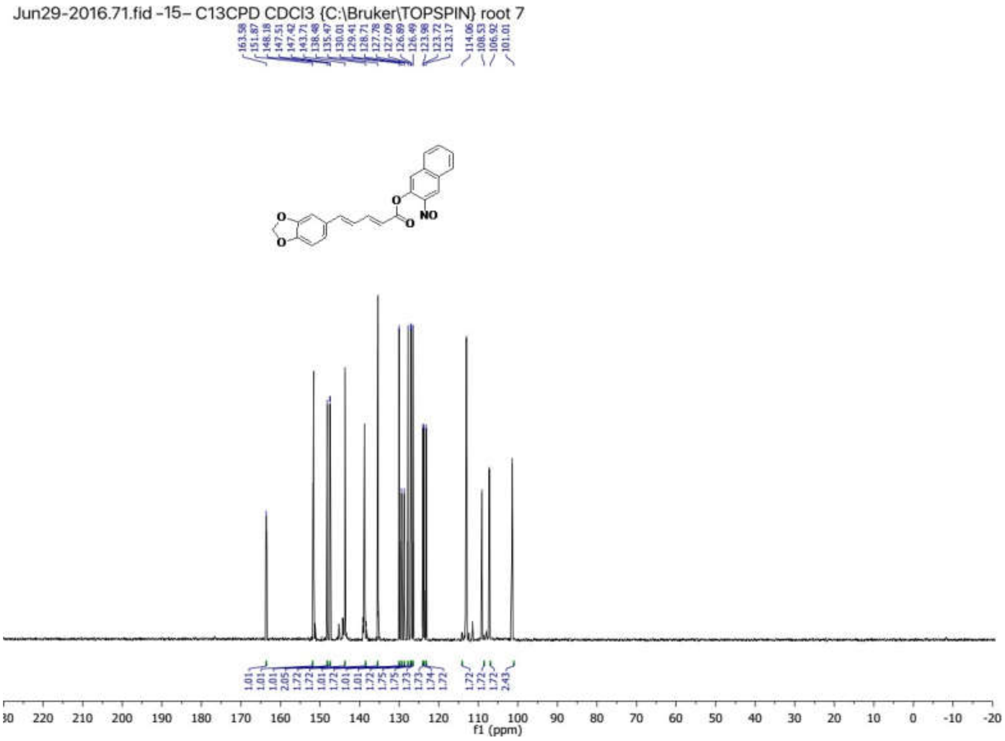

Series (IV) compound 17a IR

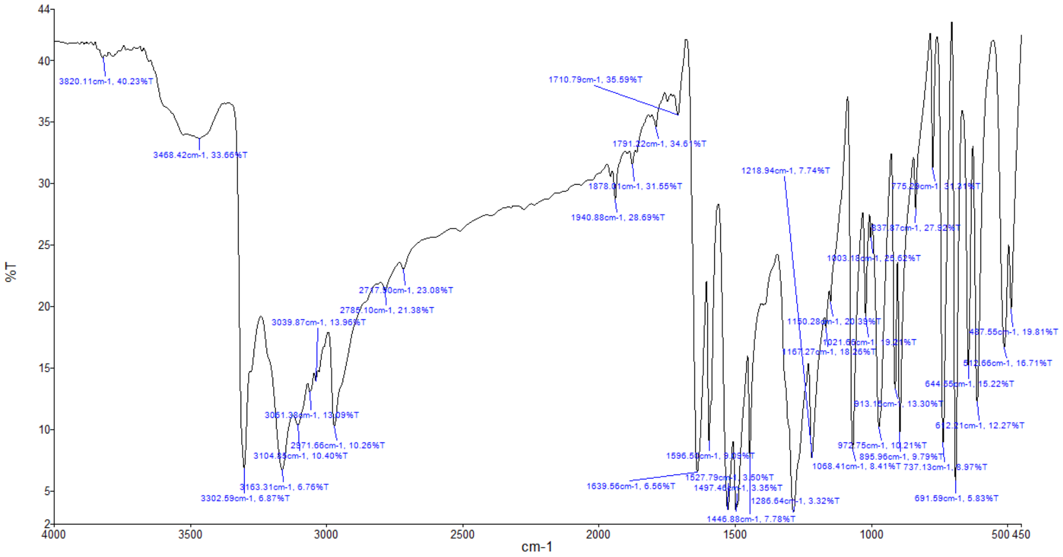

Series (IV) compound 17a ¹H-NMR

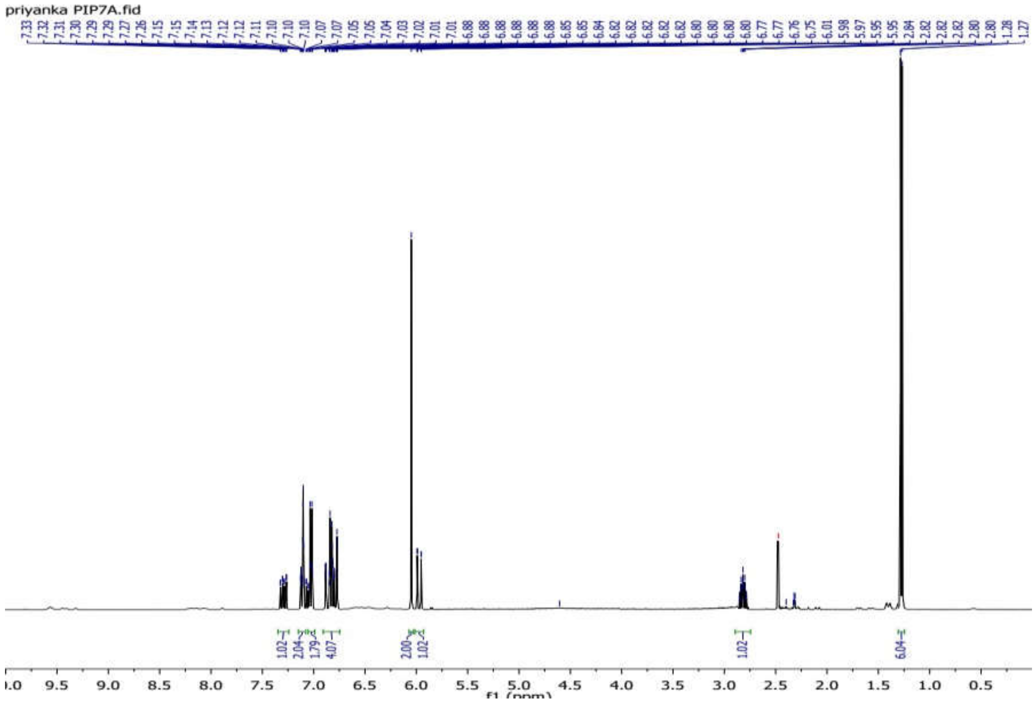

Series (IV) compound 17a ¹³C-NMR

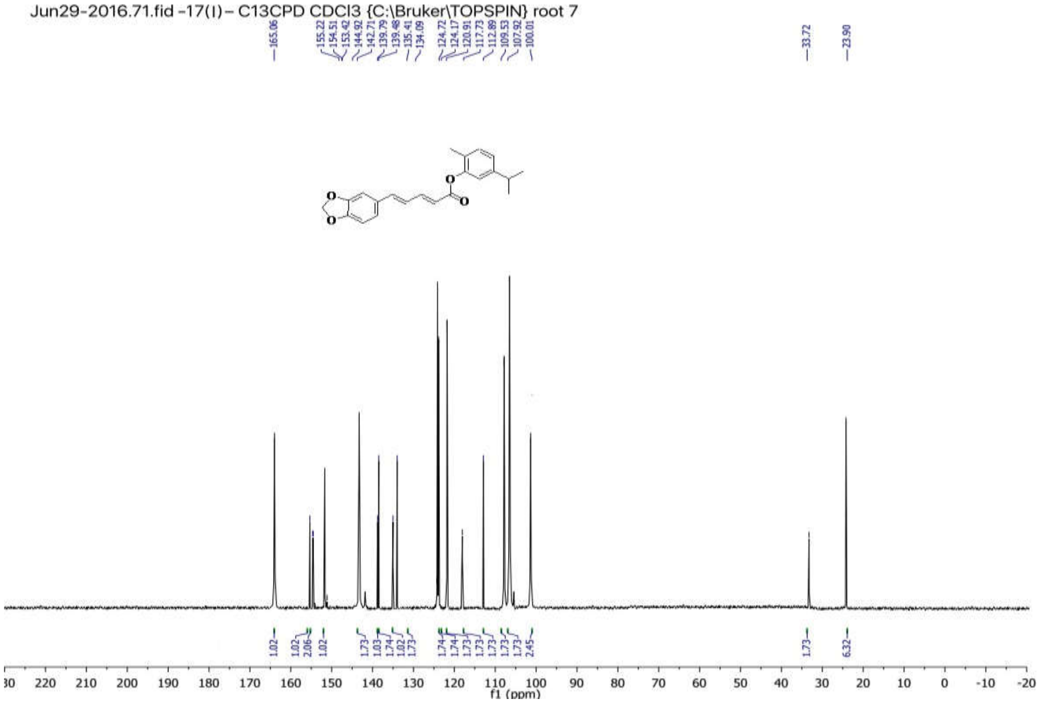

Series (IV) compound 17b IR

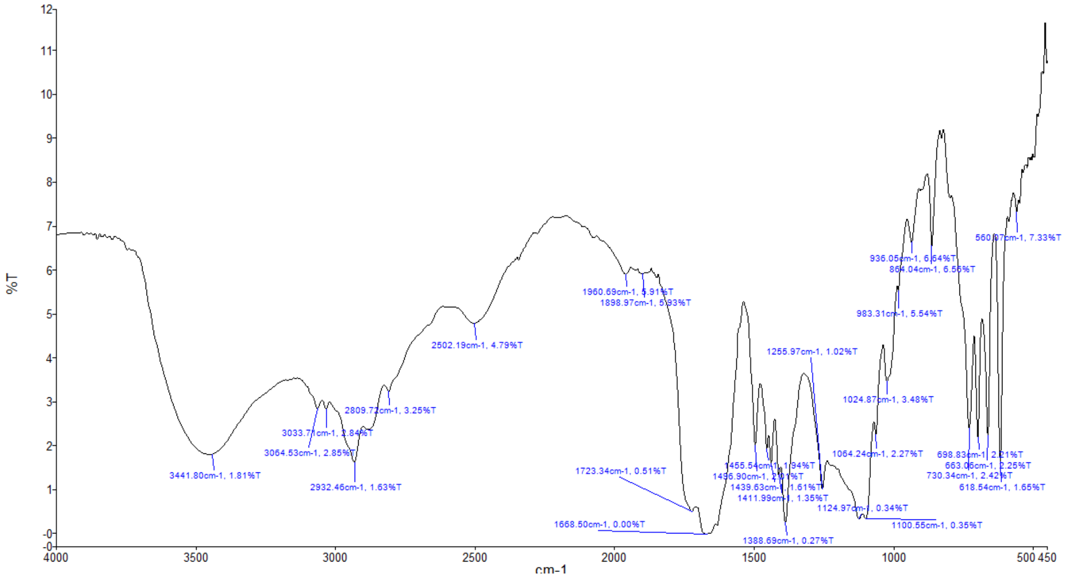

Series (IV) compound 17b ^{1}H-NMR

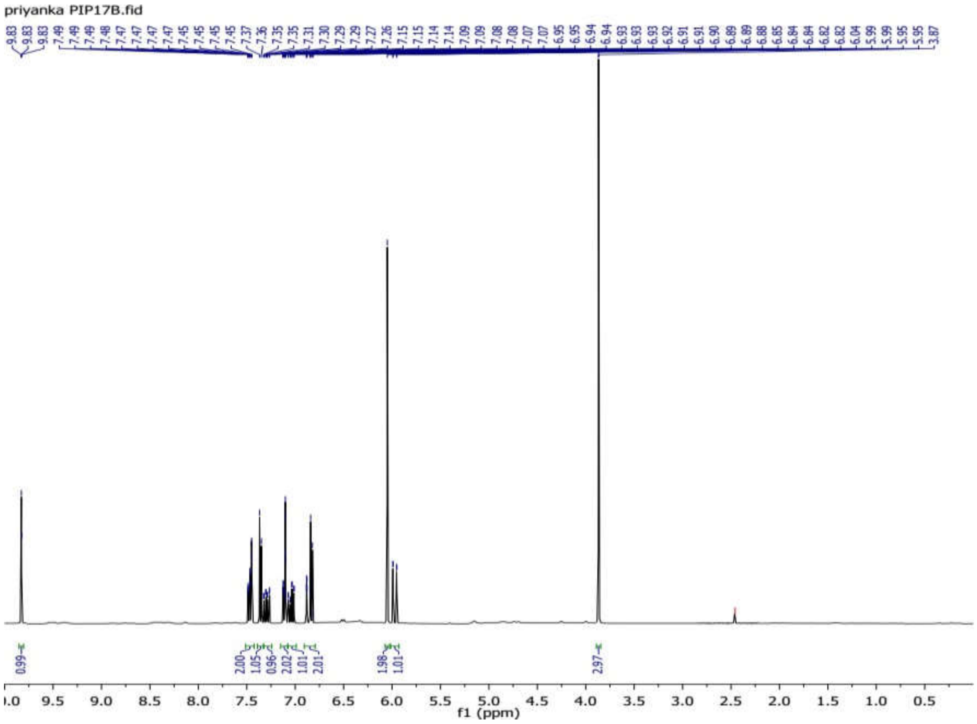

Series (IV) compound 17b ^{13}C-NMR

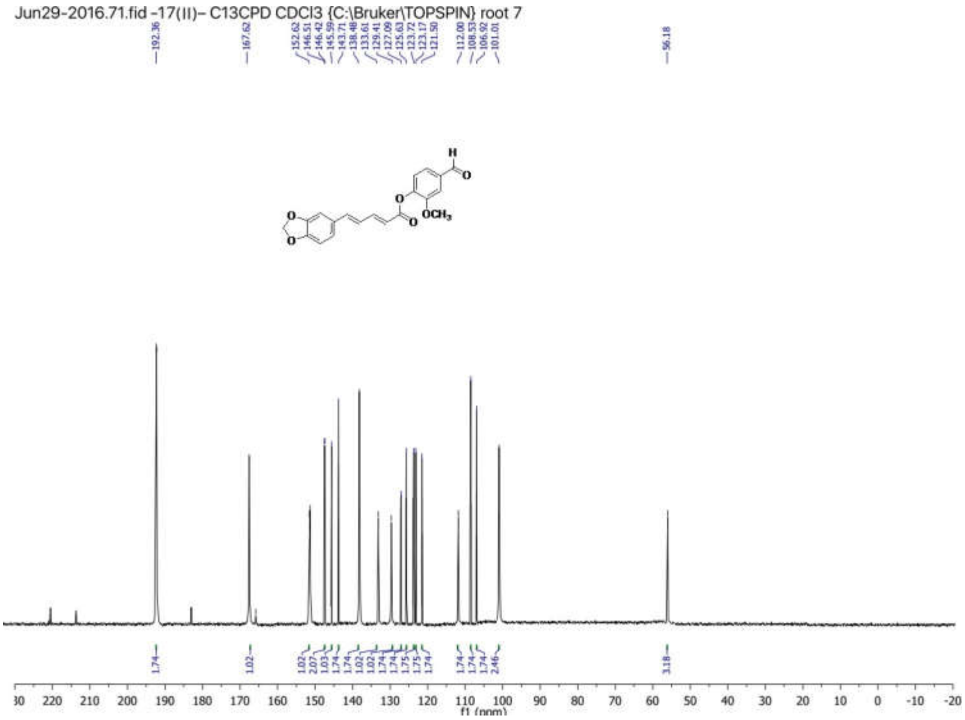

Series (IV) compound 17c IR

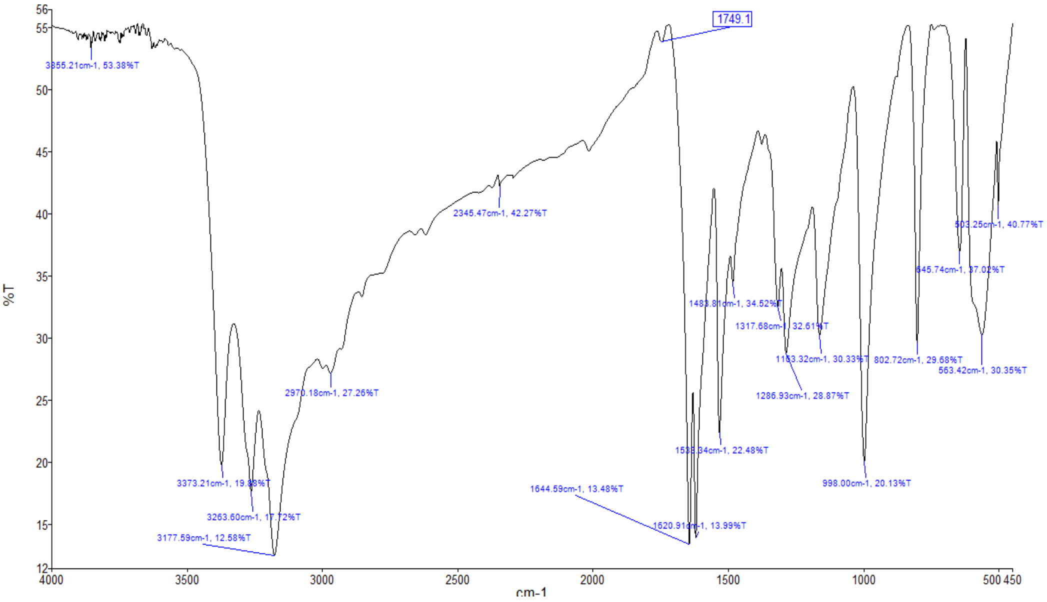

Series (IV) compound 17c ^{1}H-NMR

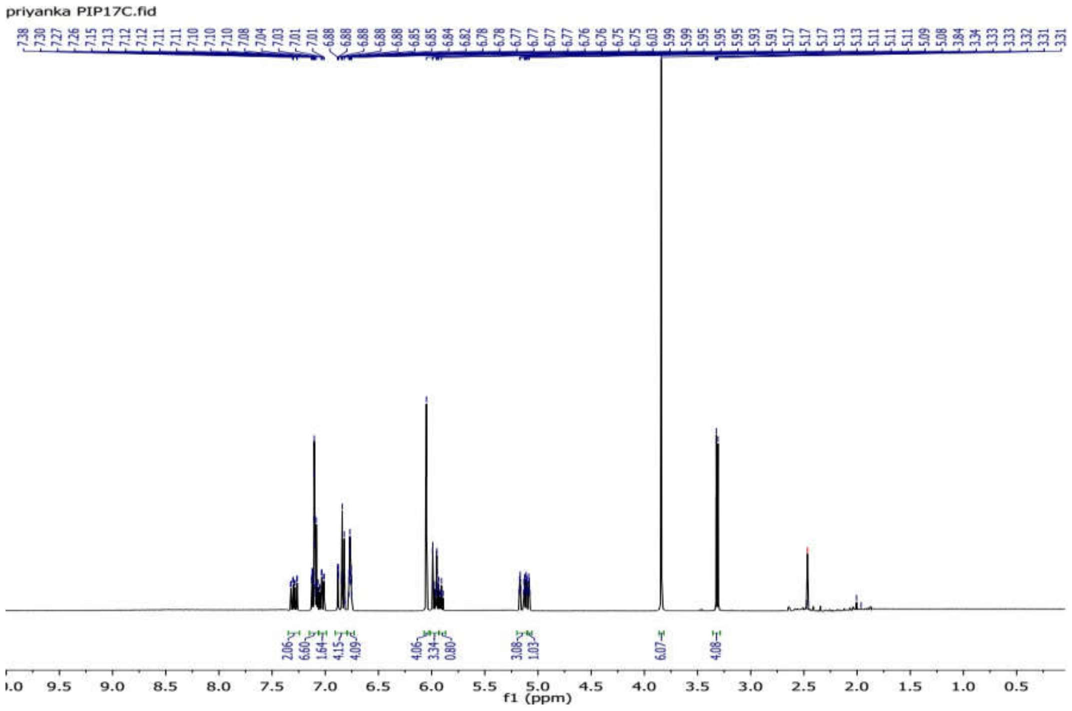

Series (IV) compound 17c ^{13}C-NMR

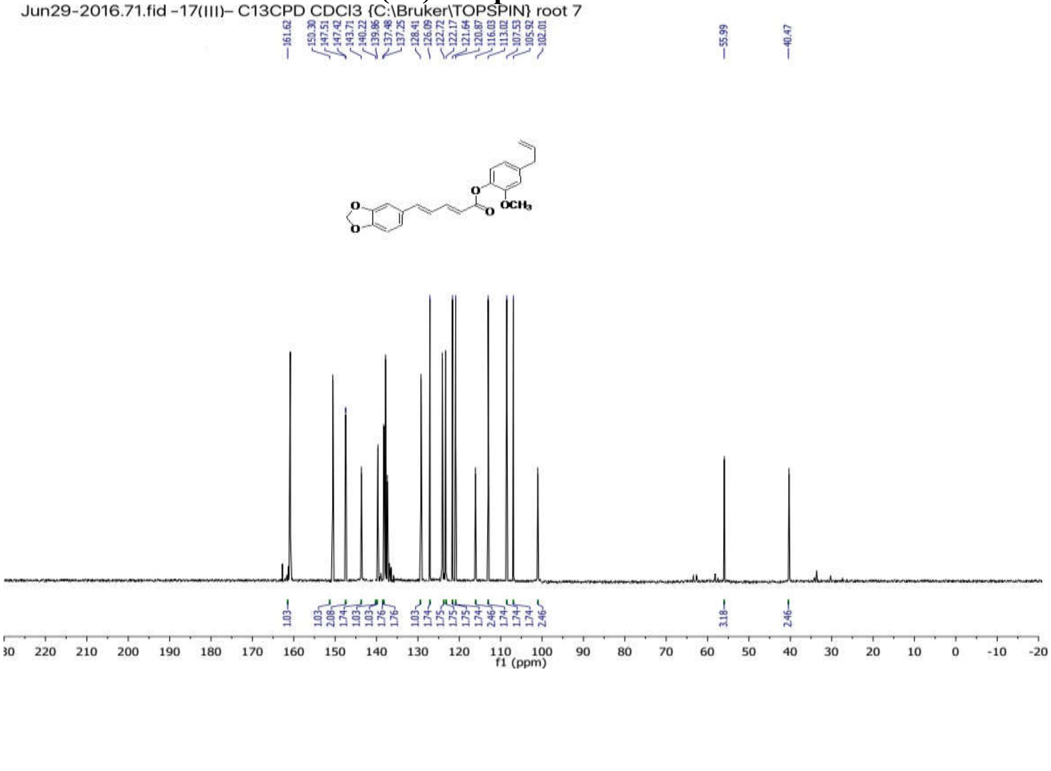

Supplement: Supplementary file 1 — Additional file 1. Spectral data of synthesised compounds. [file 13065_2020_661_MOESM1_ESM.pdf]
